# Natural variation in *CHELATASE SUBUNIT I-A* increases grain weight and enhances wheat yield

**DOI:** 10.1093/plcell/koaf212

**Published:** 2025-08-30

**Authors:** Bo Wei, Yike Liu, Mingjie Lyu, Rui Chen, Jian Ma, Changbin Yin, Ju Huang, Lixin Peng, Zhen Fu, Qilu Song, Fei Lu, Xiangqi Zhang, Yuling Jiao

**Affiliations:** State Key Laboratory of Wheat Improvement, Peking University Institute of Advanced Agricultural Sciences, Shandong Laboratory of Advanced Agricultural Sciences in Weifang, Weifang, Shandong 261325, China; Key Laboratory of Crop Molecular Breeding, Ministry of Agriculture and Rural Affairs, Hubei Key Laboratory of Food Crop Germplasm and Genetic Improvement, Institute of Food Crops, Hubei Academy of Agricultural Sciences, Wuhan, Hubei 430064, China; State Key Laboratory of Vegetable Biobreeding, Tianjin Academy of Agricultural Sciences, Tianjin 300192, China; State Key Laboratory of Vegetable Biobreeding, Tianjin Academy of Agricultural Sciences, Tianjin 300192, China; State Key Laboratory of Crop Gene Exploration and Utilization in Southwest China, Triticeae Research Institute, Sichuan Agricultural University, Chengdu, Sichuan 611130, China; The Innovative Academy of Seed Design, Chinese Academy of Sciences, Institute of Genetics and Developmental Biology, Beijing 100101, China; State Key Laboratory of Wheat Improvement, Peking University Institute of Advanced Agricultural Sciences, Shandong Laboratory of Advanced Agricultural Sciences in Weifang, Weifang, Shandong 261325, China; State Key Laboratory of Wheat Improvement, Peking University Institute of Advanced Agricultural Sciences, Shandong Laboratory of Advanced Agricultural Sciences in Weifang, Weifang, Shandong 261325, China; State Key Laboratory of Wheat Improvement, Peking University Institute of Advanced Agricultural Sciences, Shandong Laboratory of Advanced Agricultural Sciences in Weifang, Weifang, Shandong 261325, China; State Key Laboratory of Wheat Improvement, Peking University Institute of Advanced Agricultural Sciences, Shandong Laboratory of Advanced Agricultural Sciences in Weifang, Weifang, Shandong 261325, China; The Innovative Academy of Seed Design, Chinese Academy of Sciences, Institute of Genetics and Developmental Biology, Beijing 100101, China; The Innovative Academy of Seed Design, Chinese Academy of Sciences, Institute of Genetics and Developmental Biology, Beijing 100101, China; State Key Laboratory of Gene Function and Modulation Research, School of Life Sciences, Peking University, Beijing 100871, China; Peking-Tsinghua Center for Life Sciences, Center for Quantitative Biology, Academy for Advanced Interdisciplinary Studies, Peking University, Beijing 100871, China; Peking University Institute of Advanced Agricultural Sciences, Shandong Laboratory of Advanced Agricultural Sciences in Weifang, Weifang, Shandong 261325, China

## Abstract

Grain weight is a major determinant of wheat (*Triticum aestivum* L.) yield and is controlled by quantitative trait loci (QTLs) derived from natural variations. Here, we report the cloning of a major yield QTL in bread wheat that functions in the chlorophyll biosynthesis pathway. We found that the natural variation of *CHELATASE SUBUNIT I-A* (*CHLI-A*), encoding a magnesium chelatase subunit, enhances chlorophyll content and photosynthesis rate, leading to increased leaf area and grain weight. Increasing *CHLI-A* expression increases grain weight, indicating that photosynthesis is a limiting factor of wheat yield potential. The *CHLI-A* gene is absent in a portion of elite varieties, suggesting the potential of using this gene to improve grain yield in wheat.

## Teaser

Manipulating and optimizing photosynthesis-related genes to increase grain weight offers a viable strategy for sustaining crop yields.

## Introduction

Common wheat (*Triticum aestivum* L.) is one of the most staple crops in the world and provides a food supply for approximately 40% of the world's population (Shiferaw et al. 2013). With the increasing demand for food and the reduction in available arable land, continuously improving the grain yield per unit area has become a crucial goal in crop breeding. The unique domestication history of common wheat makes it a constraint to utilize natural variations in wild populations (He et al. 2019; Zhou et al. 2020). However, long-term breeding practices have generated a large reservoir of variations in landraces (Hao et al. 2020; Li et al. 2022). In particular, introgressions from tetraploid wheat and other relative species via hybridization with hexaploid common wheat have expanded the pool of natural variations (Adamski et al. 2021; Liu et al. 2021; Yan et al. 2023; Niu et al. 2023b). In recent years, many quantitative trait loci (QTLs) related to grain yield have been identified. However, only a few, such as *VEGETATIVE TO REPRODUCTIVE TRANSITION-A2* (*VRT-A2*), *CONSTANS-LIKE-B5* (*TaCol-B5*) and *Zn-F* (encoding a RING-type E3 ligase) (Liu et al. 2021; Zhang et al. 2022; Song et al. 2023), have been positionally cloned and functionally characterized. Wheat yield represents the multiplicative integration of 3 components: spike number per unit area, grain number per spike, and grain weight. Among these traits, grain weight has been one of the main target traits in breeding due to its high genetic and environmental stability (Reynolds et al. 2012; Zhang et al. 2018; Chen et al. 2020; Cheng et al. 2020; Wang et al. 2020a; Liu et al. 2021; Song et al. 2023; Niu et al. 2023b). Grain size is positively correlated with grain weight, and has also been widely used as an agronomic trait (Wei and Jiao 2024).

In general, there is a trade-off between grain weight and grain number. In addition to improving individual yield traits, optimizing photosynthesis is a promising strategy for increasing crop yield (Wei and Jiao 2024). By manipulating carbon capture and conversion, scientists have achieved remarkable increases in photosynthesis and crop yield (Ort et al. 2015; Wang et al. 2020b). Identifying new target genes from crops holds promise for better optimizing the complex photosynthesis networks. During photosynthesis, solar energy is utilized and drives the accumulation of plant biomass and sink storage, such as in crop grains (Batista-Silva et al. 2020). Recent studies using tetraploid wheat staygreen mutants have identified HEAT SHOCK PROTEIN90.2-B (HSP90.2-B), a cytosolic molecular chaperone folding photosynthetic protein (Yan et al. 2023), and ASPARTIC PROTEASE1 (APP-A1), which degrades PsbO of photosystem II (Niu et al. 2023b). These mutants also have increased grain size and weight, and hold promise for increasing yield in tetraploid and hexaploid wheat.

The natural alleles of crops are extensively selected, including by agricultural practices, and are valuable resources that minimize adverse effects in applications for breeding and agronomy. Here, we positionally cloned a grain yield-enhancing QTL in common wheat that encodes CHELATASE SUBUNIT I-A (CHLI-A), a magnesium chelatase subunit. *CHLI-A* shows presence and absence variation (PAV) in wheat landrace and elite cultivars, and introducing *CHLI-A* into a *CHLI-A* null cultivar significantly increases grain weight, and enhances the chlorophyll content and net photosynthetic rate. Importantly, overexpressing *CHLI-A* can significantly increase grain weight, suggesting its potential value in enhancing yield in wheat.

## Results

### A dominant QTL for grain weight per spike was mapped on chromosome 7A

**Figure 1.**
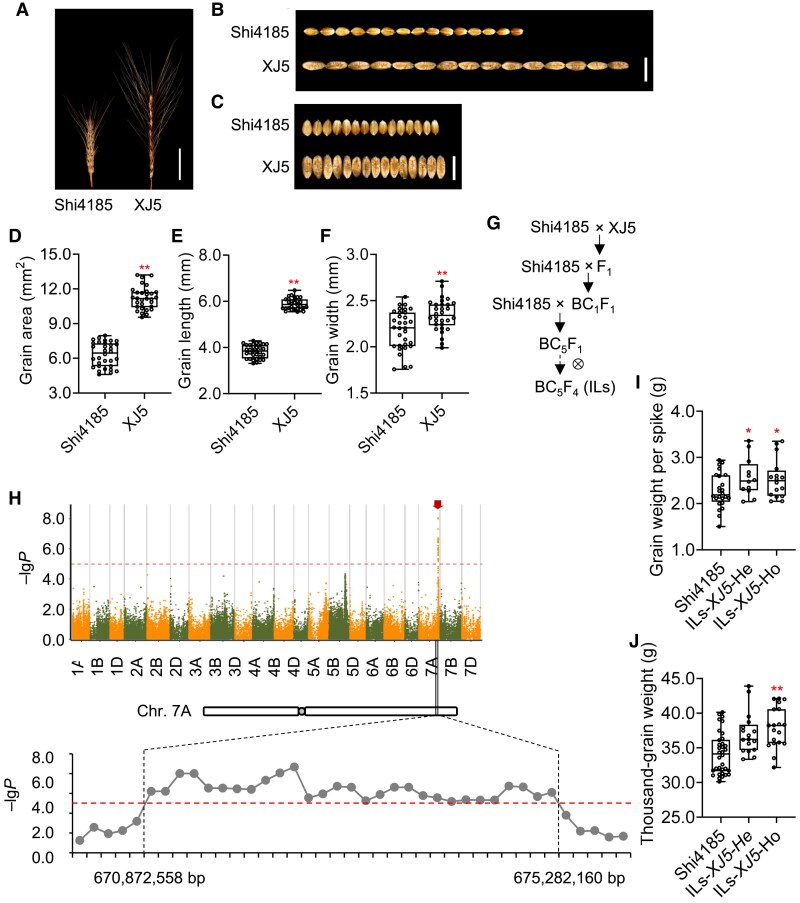
Mapping and validation of *QGwps.pku-7A*. **A)** Comparison of spike structure between cv. Shi4185 and var. XJ5. Images were digitally extracted for comparison. Scale bar, 4 cm. **B** and **C)** Phenotypic comparison of the grain length (B) and grain width (C) between cv. Shi4185 and var. XJ5. Images were digitally extracted for comparison. Scale bar, 1 cm. **D** to **F)** Statistical comparisons of the grain size (D), grain length (E), and grain width (F) between cv. Shi4185 and var. XJ5. Boxes represent interquartile range, horizontal lines denote median, whiskers = min–max, n = 30 to 32. **P < 0.01 (two-tailed Student's *t*-test). **G)** Schematic diagram for the development of introgression line (IL) population using var. XJ5 as donor parent and cv. Shi4185 as receptor parent. BC, backcross. IL, introgression line. **H)** Mapping of the *QGwps.pku-7A* was performed by association analysis using agronomic data from 2019. The upper panel shows the Manhattan plot for grain weight per spike using MLM model of GEMMA software. Negative log_10_  *P*-values (–lg*P*) of the filtered high-quality SNPs were plotted against their genomic positions. The dashed lines corresponds to the Bonferroni-corrected thresholds of –lg*P* = 5.00 (*P* = 1.00× 10^–5^). The arrow indicates the region with significant signals on chromosome 7A. The lower panel provides a zoomed-in view of the QTL and flanking regions, showing the –lg*P* value of each SNP. The points above the red line represent significant SNPs with –lg*P* ≥ 5.00. **I** and **J)** Comparative analysis of grain weight per spike (*n* = 12 to 34) (I) and thousand-grain weight (*n* = 12 to 34) (J) among pooled ILs (collectively referred to as ILs-*XJ5*) with heterozygous (He) or homozygous (Ho) introgressed *QGwps.pku-7A* and cv. Shi4185, respectively. *P < 0.05; **P < 0.01, compared with cv. Shi4185 (two-tailed Student's *t*-test).

We selected Xinjiang wheat (*T. petropavlovskyi* Udacz. et Migusch.), a Chinese endemic hexaploid wheat landrace collection with higher grain weight (Chen et al. 2013), as a tool to investigate the genetic basis of grain yield (Fig. 1A). While many *T. petropavlovskyi* varieties contain the *P1* locus associated with elongated glumes and grains (Liu et al. 2021; Xiao et al. 2021), some varieties like XJ5 exhibit a balanced increase in grain length and width (Figs. 1, B to F). To identify genes promoting grain size without affecting glume length, we crossed the *T. petropavlovskyi* variety XJ5 with cv. Shi4185, an elite common wheat cultivar with small grains (Gu et al. 2015). An introgression line (IL) population (BC_5_F_4_) was developed using cv. Shi4185 as the recurrent parent and var. XJ5 as the donor parent (Fig. 1G). The IL population displayed introgressed fragments distributed genome-wide across different lines. Reduced-representation sequencing and SNP-calling yielded 342,301 genome-wide single-nucleotide polymorphisms (SNPs) for the IL population, with introgressed exogenic DNA fragments identified through SNP comparisons (Supplementary Data Set 1).

Grain weight per spike was measured for each IL in field trials in 2 consecutive years (2018 to 2019 and 2019 to 2020) as a yield proxy. Association analysis using 101,895 high-quality SNPs from 164 IL individuals was performed with a mixed linear model (MLM), integrating phenotypic data (Supplementary Table S1). This revealed a QTL for grain weight per spike, designated *QGwps.pku-7A*, mapped to a ∼4.41-Mb interval on chromosome 7A (670.87 to 675.28 mb, Chinese Spring RefSeq v1.0), supported by 29 contiguous highly significant SNPs (*P* ≤ 10^−5^) (Fig. 1H). Among these, 4 SNPs exceeded the 1% genome-wide significance threshold (*P* ≤ 9.81× 10^−8^), while 13 reached to the 5% threshold (*P* ≤ 4.91× 10^−7^) (Fig. 1H; Supplementary Data Set 2). Comparative analysis of grain weight per spike among ILs that were homozygous, heterozygous, or null *QGwps.pku-7A* genotypes confirmed its dominant genetic effect (Fig. 1I; Supplementary Fig. S1A).

Since grain weight per spike combines grain weight and grain number per spike, we quantified both traits. Analysis of the IL population revealed that *QGwps.pku-7A*-containing ILs showed significantly higher thousand-grain weights than cv. Shi4185 (Fig. 1J; Supplementary Fig. S1B), while their grain number per spike was comparable with cv. Shi4185 (Supplementary Figs. S1, C and F). Additionally, spike number per plant and plant height did not differ between *QGwps.pku-7A*-containing ILs and cv. Shi4185 (Supplementary Figs. S1, D, E, G and H).

We focused on 3 *QGwps.pku-7A*-containing ILs (IL734, IL838, and IL915; Supplementary Figs. S2, A and B). All exhibited significantly greater grain width and length than cv. Shi4185 (Supplementary Fig. S2C), with corresponding increases in thousand-grain weights (Supplementary Figs. S2, D and G) and grain weights per spike (Supplementary Figs. S2, E and H). Conversely, their grain number per spike remained similar to cv. Shi4185 (Supplementary Figs. S2, F and I). Together, these results indicate that *QGwps.pku-7A* specifically enhances grain weight without reducing grain number, thereby increasing grain weight per spike.

### Molecular characterization of *QGwps.pku-7A*

Based on the Chinese Spring genome RefSeq v1.0, the *QGwps.pku-7A* interval contains 64 annotated genes (Fig. 1H; Supplementary Data Set 2). To assess nucleotide polymorphisms, we compared the *QGwps.pku-7A* region between cv. Shi4185 and var. XJ5 using resequencing data from this study and previous publications (Guo et al. 2020), identifying 11,780 SNPs and 847 indels (Supplementary Data Set 3). Of these, 11,563 variants resided in noncoding regions (intergenic/intronic), while 25 SNPs occurred in coding regions—15 causing missense mutations across 8 genes and 10 being synonymous (Supplementary Data Set 4).

**Figure 2.**
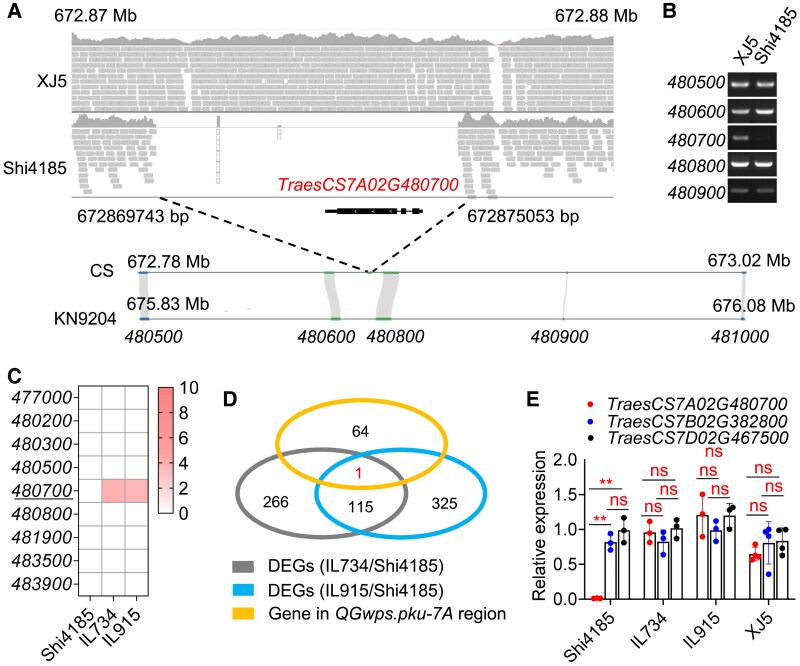
Molecular cloning of *CHLI-A*. **A)** Comparison of the *CHLI-A* genomic region between var. XJ5 and cv. Shi4185 and genomic syntenic comparison between var. Chinese Spring and cv. KN9204 reference genomes. The gray symbols are the sequencing reads mapped to the Chinese Spring genome. The dark grey bar chart indicates the coverage. Black dashed lines labeled the absent genome region in cv. Shi4185. **B)** Comparative detection of genes up and downstream of *TraesCS7A02G480700* between var. XJ5 and cv. Shi4185. **C)** Expression comparisons of all genes with missense SNP mutations or PAV (underlined) in the *QGwps.pku-7A* region in cv. Shi4185 and 2 ILs. The heatmap shows RPKM values. **D)** Venn diagram showing the candidate genes identified as satisfying differential expression among cv. Shi4185 and the ILs and located in the *QGwps.pku-7A* region. Differential expression was identified by twofold change in expression level (log_2_FC ≥ 1) and Q_value_ ≤ 0.05. **E)** The relative expression of the 3 homoeologous genes *CHLI-A* (*TraesCS7A02G480700*), *CHLI-B* (*TraesCS7B02G382800*) and *CHLI-D* (*TraesCS7D02G467500*) among cv. Shi4185, IL734, IL915, and var XJ5. The values are shown as mean ± SD of 3 to 4 biological replicates. DEGs, differently expressed genes. ns indicates nonsignificance. **P < 0.01 (two-tailed Student's *t*-test).

A critical finding was a 5,311-bp deletion (Chr7A: 672,869,743 to 672,875,053 bp) in cv. Shi4185 relative to var. XJ5, encompassing *TraesCS7A02G480700* (Chr7A: 672,872,712∼672,874,427; Fig. 2A). Comparative genomics revealed syntenic conservation in flanking regions between cv. Kenong9204 (KN9204) (Shi et al. 2022) and Chinese Spring, but *TraesCS7A02G480700* was absent in cv. KN9204 (Fig. 2A). Gene-specific PCR confirmed the presence of *TraesCS7A02G480700* in var. XJ5 (Fig. 2B), *QGwps.pku-7A*-containing ILs, and cv. Fielder (Supplementary Figs. S2, A and B), but is absence in cv. Shi4185 (Fig. 2B and Supplementary Fig. S2B).

RNA sequencing of young spikes and immature grain from cv. Shi4185, IL734, and IL915 revealed critical insights. Notably, none of the 8 genes with amino acid alternations showed detectable expression (Fig. 2C; Supplementary Datasets S5 and S6), whereas *TraesCS7A02G480700*—the gene exhibiting presence-absence variation (PAV)—displayed strong expression in both ILs (Fig. 2C). Comparative transcriptome analysis identified 115 common differently expressed genes (DEGs) between ILs and cv. Shi4185 (Supplementary Data Set 7), including only one DEG (*TraesCS7A02G480700*) located within the *QGwps.pku-7A* interval (Fig. 2D). By contrast, the homoeologous genes *TraesCS7B02G382800* and *TraesCS7D02G467500* were both present and expressed in cv. Shi4185, IL734, IL915, var. XJ5, and cv. KN9204 (Li et al. 2018) (Fig. 2E; Supplementary Fig. S3). These results strongly support *TraesCS7A02G480700*, which encodes the magnesium chelatase CHLI subunit (designated *CHLI-A*), as the causal gene underlying the *QGwps.pku-7A* locus.

### *CHLI-A* enhances grain weight and size

**Figure 3.**
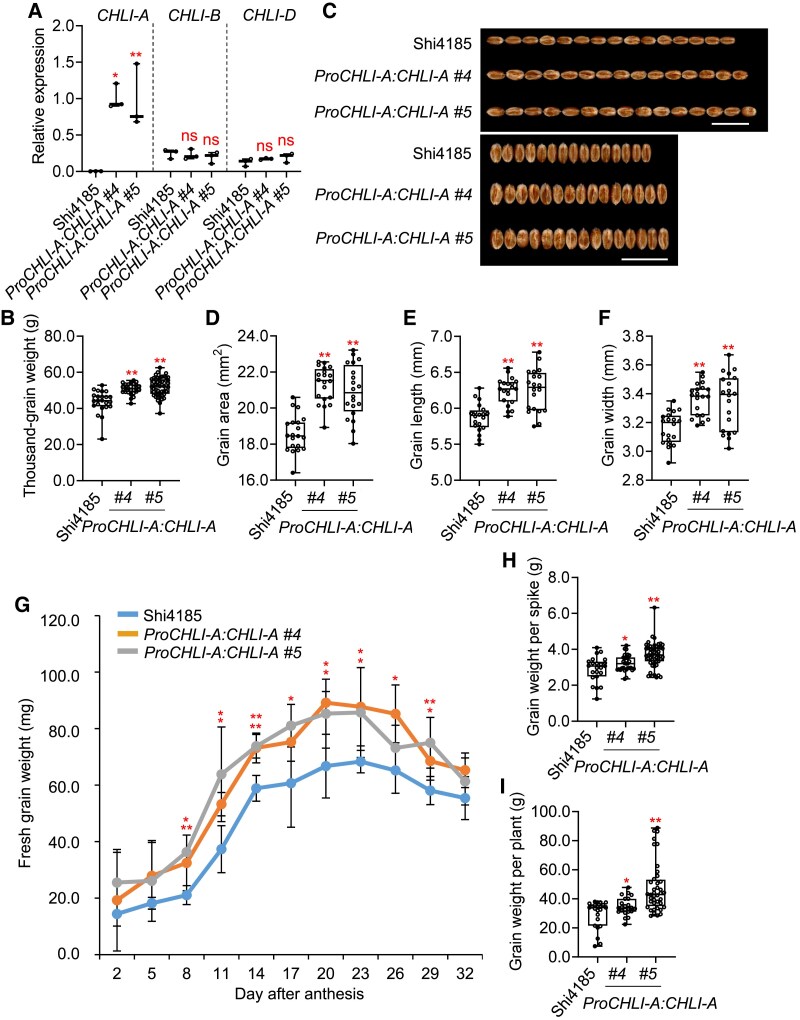
Rescuing *CHLI-A* in cv. Shi4185 can increase the grain weight and grain size. **A)** Relative expression levels of the 3 homoeologous *CHLI* genes in cv. Shi4185 and *ProCHLI-A:CHLI-A* transgenic lines are presented as means ± SD of 3 biological replicates. **B)** Comparative analysis of the thousand-grain weight between cv. Shi4185 and each *ProCHLI-A:CHLI-A* transgenic line (*n* = 23 to 51). Boxes represent interquartile range, horizontal lines denote median, whiskers = min–max. **C)** Phenotypic comparison of cv. Shi4185 and *ProCHLI-A:CHLI-A* transgenic lines. Images were digitally extracted for comparison. Scale bar, 1 cm. **D** to **I)** Comparative analysis of the grain area (*n* = 20) (D), grain length (*n* = 20) (E), grain width (*n* = 20) (F), fresh grain weight after anthesis (*n* = 3 to 5) (G), grain weight per spike (*n* = 23 to 51) (H), and grain weight per plant (*n* = 23 to 51) (I) between cv. Shi4185 and each *ProCHLI-A:CHLI-A* transgenic line. *P < 0.05; **P < 0.01indicate significant differences compared with cv. Shi4185 (two-tailed Student's *t*-test) in all statistical analyses.

To validate *CHLI-A* function, we introduced the native promoter-driven *CHLI-A* construct (*ProCHLI-A:CHLI-A*) into cv. Shi4185, generating stable transgenic lines. Two independent homozygous transgenic T_3_ lines (*#4* and *#5*), each carrying 2 transgene copies (Supplementary Table S2), were analyzed. Quantitative RT-PCR confirmed specific upregulation of *CHLI-A*, but not *CHLI-B* or *CHLI-D*, in transgenic lines (Fig. 3A). These lines exhibited significant increases in grain weight (Fig. 3B), with corresponding enhancements in grain length, width, and area (Figs. 3, C to F). Dynamic grain filling analysis revealed accelerated fresh weight accumulation in transgenic lines from 14 to 28 d post anthesis (Fig. 3G). Crucially, agronomic traits—including plant height, spike number/length, spikelet number, and grain number per spike—remained unchanged (Supplementary Figs. S4, A to G). Consequently, grain weight per spike and per plant significantly increased in transgenic lines versus cv. Shi4185 (Figs. 3, H and I).

**Figure 4.**
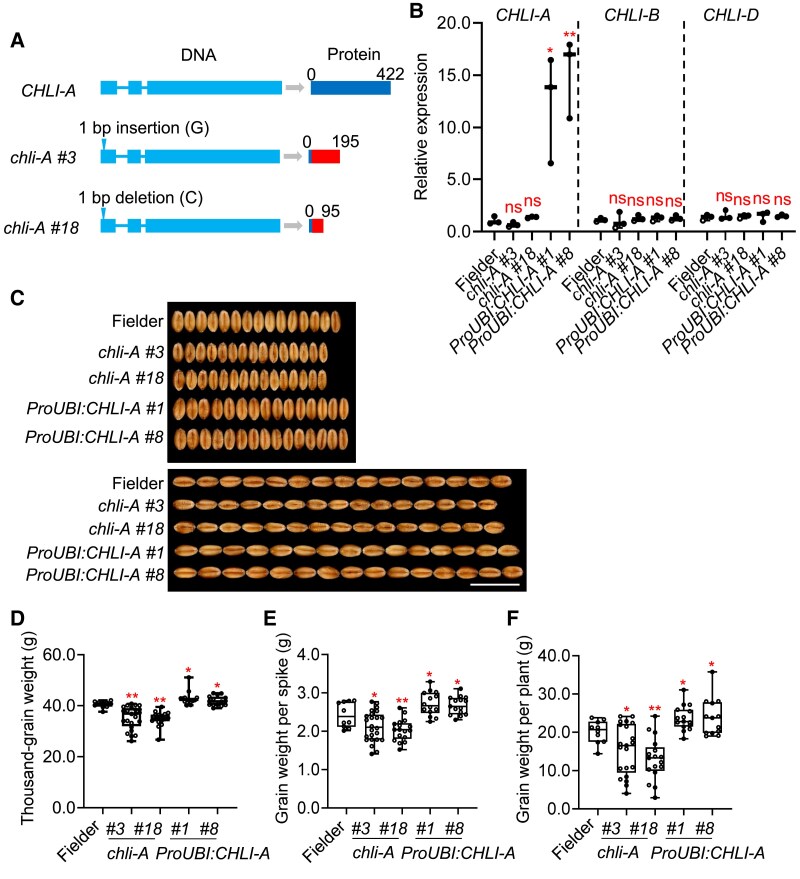
Grain weight and grain size in *CHLI-A* mutants and overexpressing lines in cv. Fielder. **A)** Construction of CRISPR/Cas9-based mutants of the *CHLI-A* gene in cv. Fielder. **B)** Comparison of relative expression levels of homoeologous *CHLI* genes among cv. Fielder, *chli-A* mutants, and *ProUBI:CHLI-A* transgenic lines. Each value represents means ± SD of 3 biological replicates. **C)** Phenotypic comparison of grain width and grain length among cv. Fielder, independent *chli-A* mutants and *ProUBI:CHLI-A* T_3_ lines harvested in field. Images were digitally extracted for comparison. Scale bar, 1 cm. **D** to **F)** Comparative analysis of the thousand-grain weight (D), grain weight per spike (E) and grain weight per plant (F) between cv. Fielder, *chli-A* mutants or *ProUBI:CHLI-A*. Boxes represent interquartile range, horizontal lines denote median, whiskers = min–max, *n* = 10 to 24. ns indicates nonsignificance. *P < 0.05; **P < 0.01 indicate significant differences compared with cv. Fielder in all statistical analyses (two-tailed Student's *t*-test).

We further engineered 2 *chli-A* mutants in cv. Fielder, which contains intact *CHLI-A* (Supplementary Fig. S2B) via single-base insertion or deletion in the first exon, generating premature stop codons (Fig. 4A). Homoeologous genes remained unaltered (Fig. 4B). Compared with cv. Fielder, mutants displayed reduced grain weight and size (Figs. 4, C and D) and decreased plant height (Supplementary Fig. S5A), while spike architecture traits remained unaffected (Supplementary Figs. S5, B to E). Complementary evidence from gain-of-function and loss-of-function approaches conclusively demonstrates *CHLI-A* as the genetic determinant of grain weight variation.

### Overexpressing *CHLI-A* further promotes grain weight

To test whether a further increase in *CHLI-A* expression can increase grain weight, we overexpressed *CHLI-A* by generating *ProUBI:CHLI-A* transgenic lines in the Fielder cultivar background. We observed a 12-fold increase in *CHLI-A* levels in the leaves of the transgenic lines, while the 2 homoeologous genes, *CHLI-B* and *CHLI-D*, showed comparable expression levels between cv. Fielder and the transgenic lines (Fig. 4B). We found that grain weight and size significantly increased in 2 independent T_3_  *ProUBI:CHLI-A* lines (Figs. 4, C and D). Similarly, the grain weight per spike, and grain weight per plant also significantly increased in the *ProUBI:CHLI-A* lines (Figs. 4, E and F). On the other hand, plant height, spike number per plant, spike length, spikelet number per spike, and grain number per spike did not change (Supplementary Fig. S5). Therefore, further increasing *CHLI-A* expression above the endogenous level is an efficient strategy for increasing grain yield.

### *CHLI-A* increases leaf size and photosynthetic efficiency

**Figure 5.**
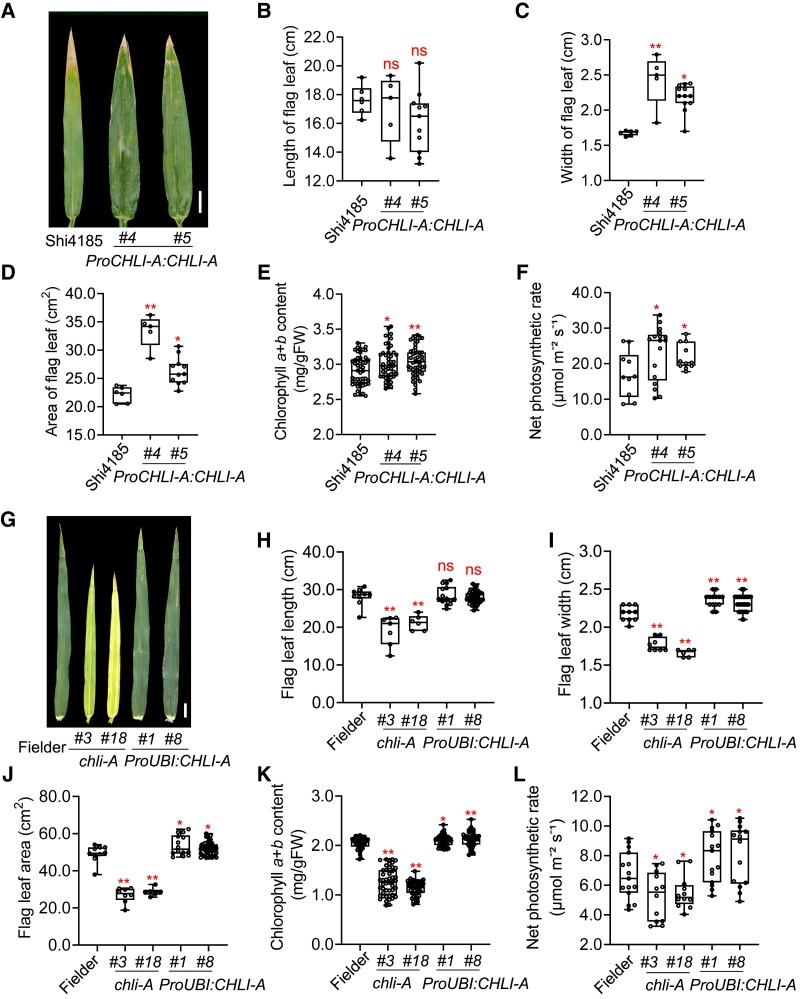
*CHLI-A* can increase leaf area, chlorophyll content and the net photosynthetic rate. **A)** Phenotypic comparison of flag leaves at the flowering stage between cv. Shi4185 and *ProCHLI-A:CHLI-A* growing in the field. Scale bar, 2 cm. **B** to **D)** Comparison of the length (B), width (C), and area (D) of flag leaves between cv. Shi4185 and each *ProCHLI-A:CHLI-A* transgenic line (*n* = 5 to 11). Boxes represent interquartile range, horizontal lines denote median, whiskers = min–max. **E** and **F)** Comparisons of the contents of chlorophyll (*n* = 47 to 51) (E) and the net photosynthetic rate (*n* = 10 to 17) (F) between cv. Shi4185 and each *ProCHLI-A:CHLI-A* transgenic line at the flowering stage in the field. ns indicates nonsignificance. *P < 0.05; **P < 0.01 indicate significant differences compared with cv. Shi4185 (Student's *t*-test) in statistical analyses in (B) to (F). **G)** Phenotypic comparison of flag leaves at the flowering stage of cv. Fielder, *chli-A* mutants and *ProUBI:CHLI-A* transgenic lines growing in the field. Images were digitally extracted for comparison. Scale bar, 2 cm. **H** to **L)** Comparative analysis of the length (*n* = 6 to 45) (H), width (*n* = 6 to 45) (I), and area (*n* = 6 to 45) (J) of the flag leaf, and the contents of chlorophyll (*n* = 45 to 49) (K) and the net photosynthetic rates (*n* = 12 to 14) (L) between cv. Fielder, *chli-A* mutants and *ProUBI:CHLI-A* at the flowering stage in the field. *P < 0.05; **P < 0.01 indicate significant differences compared with cv. Fielder (two-tailed Student's *t*-test) in statistical analyses in (H) to (L).

Magnesium chelatase, whose CHLI subunit is encoded by *CHLI-A*, catalyzes the first key step in chlorophyll biosynthesis (Gibson et al. 1995; Willows et al. 1996; Huang 2009). To this end, we analyzed photosynthesis and leaf growth. Unlike many other varieties (such as var. XJ5) cv. Shi4185 seedlings exhibit slightly yellowing, particularly before the jointing stage (Supplementary Fig. S6). This leaf color difference becomes less pronounced after the jointing stage. At both the seedling (Supplementary Figs. S6, A, E to G) and flowering stages (Fig. 5E; Supplementary Figs. S7, A and B), we observed significantly higher chlorophyll *a* and *b* contents in *ProCHLI-A:CHLI-A* transgenic lines compared with cv. Shi4185. Correspondingly, transgenic lines developed slightly darker flag leaves (Fig. 5A). Additionally, the net photosynthetic rate significantly increased in the transgenic lines (Fig. 5F). Furthermore, we found that the widths of the flag leaf increased in the *ProCHLI-A:CHLI-A* transgenic lines, although the leaf length remained unchanged or was slightly shorter (Figs. 5, A to C). An increase in leaf width led to increased leaf area in the transgenic lines (Fig. 5D). Moreover, we found that the width of the grain cavity-related organs, including the glumes, lemmas and paleas, also significantly increased in the transgenic lines (Supplementary Fig. S8).

More dramatic leaf yellowing was observed in *chli-a* mutants compared with cv. Fielder. Decreases in chlorophyll *a* and *b* contents and net photosynthetic rate were detected in *chli-A* mutants not only at seedling stage (Supplementary Figs. S7, E to G), but also at the flowering stage (Figs. 5, K and L, and Supplementary Figs. S7, C to D). We also detected higher chlorophyll contents and photosynthetic rate increases in *ProUBI:CHLI-A* lines (Figs. 5, K and L; Supplementary Figs. S7, C to G), although leaf color difference is less obvious (Fig. 5G). Note that chlorophyll contents are in general lower in cv. Fielder than in cv. Shi4185 (Figs. 5, E and K, and Supplementary Figs. S6, E to G and S7, A to G), which may account for the substantially stronger effects of *CHLI-A* in the cv. Fielder background. In the cv. Fielder background, both flag leaf width and length significantly decreased in *chli-A* mutants, while flag leaf width increased in *ProUBI:CHLI-A* transgenic lines (Figs. 5, G to I). This result in significantly reduced flag leaf areas in *chli-A* mutants and increased flag leaf areas in *ProUBI:CHLI-A* lines (Fig. 5J).

It should be noted that cv. Shi4185, although lacks *CHLI-A*, has higher chlorophyll *a* and *b* contents and photosynthesis rate than cv. Fielder. Chlorophyll contents and photosynthesis rate are highly variable among wheat varieties, even in model cultivar (Saeed et al. 2016). We found that cv. Shi4185 is among those with high chlorophyll contents and photosynthesis rate, whereas cv. Fielder is among those with low levels. This basal difference is associated with the stronger effects of *CHLI-A* on leaf color.

To determine whether photosynthesis is broadly affected by *CHLI-A*, we compared the expression levels of genes encoding proteins involved in the photosynthetic pathway, including *LIGHT-HARVESTING CHLOROPHYLL A/B-BINDING1* (*TaLHCB1*), *PHOSPHOENOLPYRUVATE CARBOXYLASE* (*TaPEPC*), and *RIBULOSE-1,5-BISPHOSPHATE CARBOXYLASE SMALL SUBUNIT* (*TaRBCS*). We found that the expression levels of *TaLHCB1-A*, *TaLHCB1-B*, *TaPEPC-A*, *TaPEPC-B*, *TaPEPC-D*, *TaRBCS-B* and *TaRBCS-D* were significantly higher in *ProCHLI-A:CHLI-A* lines than in cv. Shi4185. In the cv. Fielder background, comparable increases were observed in *ProUBI:CHLI-A* transgenic lines, while the expression levels of these genes were reduced in *chli-A* mutants (Supplementary Figs. S9 and S10).

### *CHLI-A* increases grain yield in the field

**Figure 6.**
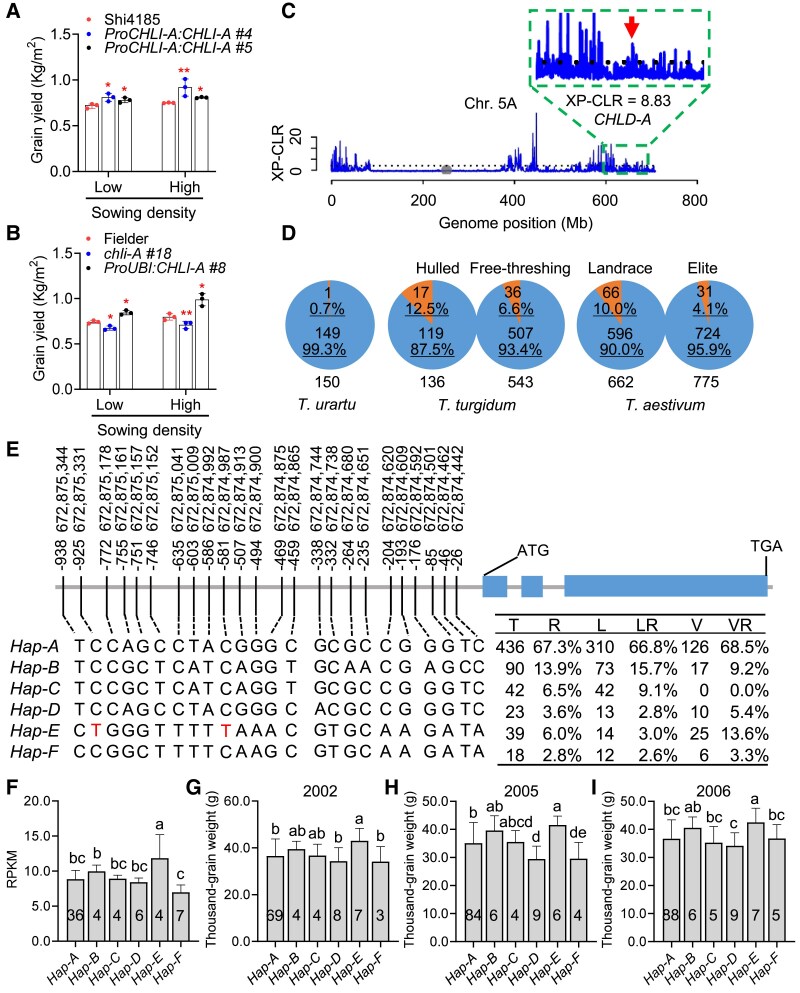
Selection of *CHL* genes for wheat domestication and breeding. **A** and **B)** Comparison of grain yields in the field with 2 levels of sowing density. The data are shown as mean ± SD (*n* = 3). Significance levels are determined from two-tailed Student's *t-*test. *P < 0.05; **P < 0.01. **C)** Detection of selection signature at the *CHLD-A* gene during domestication (wild einkorn vs. domesticated einkorn) using the XP-CLR method. The criterion for the top 5% of regions, i.e. XP-CLR = 3.082, is indicated by the dotted line. The arrow indicates the position of *CHLD-A* (XP-CLR = 8.83). The *x* axis is the genome position of chromosome 5A, and the *y* axis is cross-population composite likelihood ratio. **D)** Distributions of PAVs of *CHLI-A* in *T. urartu* population, *T. turgidum* populations and *T. aestivum* varieties. The numbers represent the numbers of varieties. **E)** Haplotype analysis of the *CHLI-A* gene. The upper numbers indicate the physical positions of SNPs identified within the 1-kb region upstream of the *CHLI-A* start codon (Chinese Spring RefSeq v1.0). The lower numbers denote SNP positions relative to the *CHLI-A* start codon. SNPs are shown in the bottom, with *Hap-E* variants highlighted. The table on the right summarizes haplotype statistics. T, total accessions per haplotype; R, haplotype frequency; L and V, counts of landrace and modern varieties, respectively, containing corresponding haplotype; LR and VR, proportions of landrace and modern varieties, respectively, having the corresponding haplotype. **F)**  *CHLI-A* expression levels across haplotypes. Expression values (RPKM: reads per kilobase per million mapped reads) are compared between haplotypes. **G** to **I)** Thousand-grain weight associations with haplotypes. Thousand-grain weight values for 6 haplotypes in 2002 (G), 2005 (H), and 2006 (I) are shown (Jiang et al. 2011; Su et al. 2011). The data are shown as mean ± SD. The number in each histogram represents the number of varieties having relevant *CHLI-A* haplotype. Bars labeled with distinct lowercase letters (a, b, c) indicate statistically significant differences (*P* < 0.05, one-way ANOVA with Dunnett's multiple comparison test); shared letters denote nonsignificance.

We subsequently tested the contribution of *CHLI-A* to field performance at the population level. We applied grain yield trials at 2 sowing densities: low density at 179 grain/m^2^, which is commonly used in Australia, Canada and the US, and high density at 536 grain/m^2^, commonly used in China (Wang et al. 2022b). The grain yield of the *ProCHLI-A:CHLI-A* lines was significantly greater than that of cv. Shi4185 at both sowing densities, and the increase was mainly attributed to the marked increase in thousand-grain weight rather than grain number per spike and spike number per unit area (Fig. 6A; Supplementary Figs. S11, A to C). Compared with those of cv. Fielder, the *ProUBI:CHLI-A* overexpressing line presented greater grain yield, and the *chli-A* mutant line had significantly lower grain yield at both sowing densities, and the changes were mainly attributed to thousand-grain weight (Fig. 6B; Supplementary Figs. S11, D to F). Overall, *CHLI-A* expression levels positively correlated with grain yield in the field trials.

### Selection of *CHL* genes for domestication and breeding

Beneficial loci are often under human selection during domestication or breeding. In common wheat, we identified 3 *CHLI* homoeologous genes on 7A, 7B and 7D which exhibited comparable levels of expression (Figs. 2, E and 4B). To test whether *CHLI* genes have been under human selection, we utilized resequencing data from a global collection of 91 diploid *Triticum* accessions (AA taxa), 121 tetraploid accessions (AABB taxa) and 172 hexaploid accessions (AABBDD taxa) to scan for selection. The cross-population composite likelihood ratio (XP-CLR) analysis could not detect genomic signatures of positive selection on *CHLI* genes (Supplementary Data Set 8). In addition to CHLI, magnesium chelatase has 2 other subunits, CHLD and CHLH. We also performed XP-CLR analysis, and identified strong selection of *CHLD-A* during the process from wild einkorn to domesticated einkorn (Fig. 6C; Supplementary Data Set 8).

To better understand the utilization of *CHLI-A* in domestication and breeding, we screened 662 landrace and 775 elite common wheat cultivars via PCR-based assays. *CHLI-A* was present in 90.0% of the landraces and 95.9% of the elite cultivars, indicating that *CHLI-A* has been broadly utilized (Fig. 6D). Nevertheless, *CHLI-A* is relatively scarce in the main wheat-producing area of North China, such as Hebei Province, where ∼25% of the cultivars lack *CHLI-A* (Supplementary Fig. S12). We also screened 150 *T. urartu* accessions and 679 *T. turgidum* accessions, including hulled and free-threshing domesticated tetraploid wheat accessions. We found only one *T. urartu* accession lacking *CHLI-A*, suggesting that the deletion of the only *CHLI* gene might have strong negative effects (Fig. 6D; Supplementary Fig. S13), which was consistent with the lethality of barley *chli* mutant (Hansson et al. 1999) and albino phenotype of the Arabidopsis *chli1 chli2* double mutant without detectable chlorophylls (Huang 2009). Among the tetraploid accessions, 90.6% had *CHLI-A* (Fig. 6D). Among them, 87.5% of hulled tetraploid wheat, which represents early-stage domestication, possessed the *CHLI-A* gene, whereas 93.4% of free-threshing tetraploid wheat, which was further selected, possessed this gene, suggesting that the presence of the *CHLI-A* gene might have been selected during the domestication of tetraploid wheat (Fig. 6D).

To further investigate natural variations of *CHLI-A* among hexaploid wheat accessions, the coding region and the 1 kb promoter region upstream of the *CHLI-A* start codon were analyzed for sequence polymorphism using the resequencing data of 648 worldwide wheat accessions (http://wheat.cau.edu.cn/WheatUnion/) (Cheng et al. 2019; Hao et al. 2020; Walkowiak et al. 2020; Zhou et al. 2020; Liu et al. 2021; Schulthess et al. 2022; Yang et al. 2022; Niu et al. 2023a). *CHLI-A* absent accessions were excluded. No SNPs were found in the coding region; however, 24 SNPs were identified in the promoter region, which distinguished the accessions into 6 haplotypes (*Hap-A* to *Hap-F*) (Fig. 6E). Transcriptome analysis revealed that *Hap-E* exhibited the highest expression level, while accessions containing *Hap-F* had the lowest level of expression (Fig. 6F) (Wang et al. 2017). Furthermore, wheat accessions with *Hap-E* presented the heaviest thousand-grain weight over 3 years (2002, 2005, and 2006) (Jiang et al. 2011; Su et al. 2011), whereas accessions containing *Hap-A, Hap-D* and *Hap-F* had lighter thousand-grain weight. *Hap-B* and *Hap-C* accessions possessed lower grain weight than *Hap-E*, but the differences were not significant (Figs. 6, G to I).

Further analysis of the wheat accessions found that *Hap-A* was the most abundant allele (67.3%), while *Hap-B* accounted for 13.9%, and *Hap-C* to *Hap-F* accounted for less than 7.0% (Fig. 6E). Among these, only the frequency of *Hap-E* substantially increased during the breeding process, from 3.0% in landraces to 13.6% in modern varieties (Fig. 6E). Therefore, *Hap-E* could be used as an elite *CHLI-A* allele for improving wheat grain yield by increasing grain weight and can be specifically monitored by the 2 C-T SNPs at positions 672,874,987 and 672,875,331 bp (Fig. 6E).

## Discussion

Increasing grain yield is an ultimate goal of agriculture, but field grain yield is a complex trait strongly influenced by both the genome and the environment. Notably, mutual antagonism among the 3 yield-related traits often constrains the final increase in grain yield in the field (Xie and Sparkes 2021). For instance, increasing grain number per spike is often shadowed by a decrease in grain weight, and *vice versa*. Ectopic expression of *TaBG1* leads to a larger grain size but exhibits a trade-off in grain number per plant, resulting in no significant overall increase in grain yield (Milner et al. 2021). On the other hand, mutants of *BH^t^/WFZP-A* and *WFZP-D* present significantly increased grain number per spike but reduced the grain weight and even decreased grain yield (Dobrovolskaya et al. 2014; Poursarebani et al. 2015; Koppolu and Schnurbusch 2019; Du et al. 2021; Li et al. 2021; Abbai et al. 2023). To fully explore the genes underlying field grain yield, we performed linkage analysis of natural varieties to identify QTLs for field grain yield improvement. To simplify our analysis, we initially focused on grain weight per spike. We introgressed genomic fragments from endemic Xinjiang landrace var. XJ5 with large grain into elite cv. Shi4185 to construct an IL population (Fig. 1G) and identified a QTL *QGwps.pku-7A* for promote grain weight per spike in cv. Shi4185 background (Fig. 1H. Field trials suggested the improvement of grain yield using those ILs, which encouraged us to further map the causal gene. QTL analysis revealed an absence of *CHLI-A* gene in cv. Shi4185 (Fig. 2). And the introduction of *CHLI-A* into cv. Shi4185 and the knockout of *CHLI-A* in cv. Fielder confirmed the function of *CHLI-A* in increasing the thousand-grain weight and grain weight per spike (Figs. 3 and 4).

Overcoming the trade-off between grain weight and grain number is one of the crucial steps in synergistically improving grain yield. Field trials indicated that the existence of *CHLI-A* positively correlated with grain weight, and had no trade-off on grain number and spike number per unit area, leading to robust overall increases in grain yield per unit area at the population level (Fig. 6; Supplementary Fig. S11). The increase was consistently found at 2 very different sowing densities, suggesting that *CHLI-A* might be broadly useful in different wheat production areas worldwide.

*CHLI-A* encodes the CHLI subunit of magnesium chelatase, which is a key enzyme that chelates Mg^2+^ into protoporphyrin IX during the biosynthesis of chlorophyll (Gibson et al. 1995; Willows et al. 1996; Huang 2009). Missing *CHLI-A* leads to reduced chlorophyll content and photosynthesis in *chli-A* mutants in cv. Fielder at the seedling stage (Figs. 5, K and L; Supplementary Figs. S7, E and F). Conversely, introducing *CHLI-A* into cv. Shi4185 (Figs. 5, E and F; Supplementary Figs. S6, E to G; S7, A and B) or overexpressing *CHLI-A* in cv. Fielder (Figs. 5, K and L; Supplementary Figs. S7, A to E) increases chlorophyll content and photosynthesis. These observations are consistent with the lower chlorophyll content observed in *CHLI* gene single-nucleotide polymorphism mutants in rice, wheat, and soybean (Zhang et al. 2015; Du et al. 2018). Furthermore, the presence of *CHLI-A* leads to increased leaf areas and enlarged grain cavity-related organs, including glumes, lemmas, and paleas (Fig. 5; Supplementary Fig. S8). The *CHLI-A* level may influence different sink dynamics. While the *CHLI-A* level is positively correlated with grain yield over a wider range, a lack of *CHLI-A* also affects storage sinks, as reflected by decreased plant height and flag leaf length in mutants. We speculate that other photosynthesis promoting loci in cv. Shi4185 alleviates the need of *CHLI-A*, as shown by the milder yellowish phenotype in cv. Shi4185 than in the *chli-A* mutants in the cv. Fielder background (Figs. 5, A and G). In fact, cv. Shi4185 has higher levels of chlorophyll *a* and *b* contents and photosynthesis rate than cv. Fielder (Figs. 5, E and F, K-L and Supplementary Fig. S7).

Photosynthesis is the intrinsic driver of biomass production and sink accumulation (Batista-Silva et al. 2020), and optimizing photosynthesis has been considered a promising strategy for increasing grain yield (Reynolds et al. 2012 ). However, the plethora of photosynthesis-related genes makes it difficult to identify efficient breeding targets. Recently, by screening staygreen mutants in tetraploid wheat, several genes encoding proteins that indirectly regulate photosynthesis have been identified and shown to increase grain yield in common wheat (Chen et al. 2020; Yan et al. 2023; Niu et al. 2023b). The overexpression of *DEHYDRATION RESPONSIVE ELEMENT BINGDING1C* (*OsDREB1C*) also increases photosynthesis, the chloroplast number, and the grain yield in wheat and rice (Wei et al. 2022). In this study, we identified a natural variation-related breeding target for effective photosynthesis enhancement and field grain yield improvement with minimal adverse effects on other agronomic traits.

Population genetic analysis indicated that *CHLD-A*, encoding the other AAA + subunit, was under selection during the domestication of einkorn (Fig. 6C; Supplementary Data Set 8). However, we could not detect selection at *CHLI-A* or the 2 homoeologous *CHLI* genes. A recent study showed that *CHLI-D* missense mutant led to strongly reduced growth and photosynthesis (Wang et al. 2024), suggesting deleterious effects. On the other hand, the role of *CHLI-A* is milder and fine-tunes photosynthesis and grain development. Given that *CHLI-A* is present in 90.0% of analyzed landraces, including Indian dwarf wheat (*T*. *aestivum* ssp. *sphaerococcum*), which occupies a basal phylogenetic position (Zhou et al. 2020), it is likely that the loss of *CHLI-A* in a subset of varieties occurs later in an adaptation to human selection. Haplotypes containing *CHLI-A* were enriched in landrace and elite modern wheat varieties, consistent with its superior agronomic performance (Figs. 6, D and E; Supplementary Fig. S12). However, *CHLI-A* is still absent in a portion of elite varieties, such as those cultivated in North China (Supplementary Fig. S12B), suggesting its potential for further utilization in breeding programs. Importantly, we found that further overexpression of *CHLI-A* in a *CHLI-A*-containing background could still increase field grain weight. Field trials supported this finding, showing an increase in yield without negative effects (Fig. 6B; Supplementary Figs. S11, D to F). Therefore, *CHLI-A* represents a promising target for further increasing grain yield during breeding (Fig. 6). Strategies such as increasing *CHLI-A* copy number or identifying/creating haplotypes with higher expression levels are efficient ways to enhance grain yield. Indeed, we identified a haplotype, *Hap-E*, which is associated with higher *CHLI-A* expression levels and increased grain weight (Figs. 6, E to I), highlighting its potential application in breeding programs. Additionally, *CHLD* and *CHLH* genes may also play roles in grain-related traits, warranting further investigation in future studies.

## Materials and methods

### Plant materials and growth conditions

Using Chinese endemic hexaploid wheat accessions, with Xinjiang wheat (*T. aestivum* ssp. *petropavlovskyi*) XJ5, as the donor parent and cv. Shi4185 as the receptor parent, we developed 3 primary IL populations (BC_2_F_3_) (Gu et al. 2015). Furthermore, the primary IL populations were randomly selected for backcrossing with the recurrent parent cv. Shi4185. After another 3 rounds of backcrossing and 4 generations of selfing, a set of introgression lines (ILs, BC_5_F_4_) were developed, including 418 independent lines.

To validate the function of *CHLI-A* gene, cv. Shi4185 was used to rescue the *CHLI-A* gene. A wheat cultivar, Fielder, having the *CHLI-A* gene in the chromosome 7A was used for the *CHLI-A* overexpression and gene knockout. A global collection of 91 diploid *Triticum* accessions (AA taxa), 121 tetraploid accessions (AABB taxa) and 172 hexaploid accessions (AABBDD taxa) were used for the cross-population composite likelihood ratio (XP-CLR) analysis of *CHLI*, *CHLD* and *CHLH*. In addition, 150 *T. urartu* accessions, 679 *T. turgidum* accessions, 662 landrace and 775 elite common wheat cultivars were used to analyze the domestication and breeding of *CHLI-A* gene. To investigate the haplotypes of the *CHLI-A* gene, a panel of 648 worldwide wheat accessions with DNA resequencing data was used (http://wheat.cau.edu.cn/WheatUnion/) (Cheng et al. 2019; Hao et al. 2020; Walkowiak et al. 2020; Zhou et al. 2020; Liu et al. 2021; Schulthess et al. 2022; Yang et al. 2022; Niu et al. 2023a). The published RNA sequencing data and thousand-grain weight over 3 years (2002, 2005, and 2006) of the mini-core collection of Chinese wheat were used for comparisons (Jiang et al. 2011; Su et al. 2011; Wang et al. 2017).

All the agronomic trait was measured in field. Before planting, 750 kg ha^−1^ compound fertilizer (15-N, 15-P_2_O_5_, 15-K_2_O) was applied as a basal dressing and 112.5 kg N ha^−1^ was applied at the jointing stage as top dressing. Irrigations of 300 and 450 m^3^ ha^−1^ water were applied at the green up stage and stem extension stage respectively. Appropriate chemicals were used to control pests and weeds. For RNA-seq, cv. Shi4185, IL734 and IL915 plants were grown in a greenhouse under long-day conditions (16 h light/8 h dark) with a light intensity of 350 *μ*mol m^−2^ s^−1^ at 25 °C and a relative humidity of 65% to 70%.

### Agronomic trait evaluation

For agronomic trait evaluation, all ILs were planted in the experimental field during both 2018 to 2019 and 2019 to 2020 cropping seasons in Changping (39° 55′ N, 116° 23′ E; Beijing, China). The *CHLI-A* transgenic lines and mutants were planted in the field during the 2022 to 2023 cropping season in Weifang (36° 50′ N, 119° 41′ E; Shandong, China). Each line was planted in a 2.0-m four-row plot with row spaced 0.25 m apart and 40 seeds per row. To avoid margin effects, about 10 individual plants in the 2 middle-lines were selected randomly for agronomic trait evaluation. The grain weight per spike and grain number per spike were measured by using the main spike of the 10 randomly selected individual plants. The thousand-grain weight was calculated by counting 3 random 100-grain of each selected line. The spike number per plant, plant height and flag leaf morphology were measured manually before harvest in field. The grain length and grain width were detected by using the seed detector (Microteck scanner MRS-9600TFU2L, Zhongjing Technology).

For the field yield evaluation, the cv. Shi4185, Fielder, *CHLI-A* transgenic lines and mutants were hand planted in a plot of 3.38 m^2^ (1.50 m × 2.25 m, 15-row plots of 1.50 m in length with 0.15 m row space) with seedling rate of approximately 179 grains/m^2^ for low density and approximately 536 grains/m^2^ for high density, respectively, in the 2022 to 2023 growing season at experimental station in Weifang. The cv. Shi4185 and IL812 were planted with close planting densities of 536 grains/m^2^ in plots of 10.80 m^2^ (1.20 m × 9.00 m, 8-row plots of 9.00 m in length with 0.15 m row space) in 2021 to 2022 and 2022 to 2023 growing seasons at the experimental station in Zhaoxian (37° 37´ N, 114° 36´ E; Hebei, China). All of the yield trials were arranged 3 replicates. For each plot, different wheat cultivars with comparable plant height were planted around the plots to avoid the margin effect which were harvested before the grain yield measurement. For the agronomic trait evaluation at population level, the plants within the field plots were all harvested for final yield comparison. Thousand-grain weight was calculated by selecting 10 random 500-grain samples in each sample. Grain number per spike was counted manually from 10 randomly selected main spikes in each plot. Spike number per unit area was calculated by counting all the spikes within a randomly selected 1-m-long row, and 10 replicates in each plot were carried out.

### Construction of the reduced representation libraries and sequencing

The young leaves of 8 to 10 seedlings were sampled and pooled for genomic DNA extraction using a modified CTAB-phenol‒chloroform method (Murray and Thompson 1980), and the DNA was quantified using a NanoDrop 2000 spectrophotometer (Thermo Fisher Scientific). Then, the high-quality genomic DNA of each IL and the parents was digested with the restriction endonuclease *Hae* III (New England Biolabs). Fragments ranging from 450 to 480 bp were excised from the gels. Sample quantification was conducted by a Qubit 4 fluorometer (Thermo Fisher Scientific), and at least 300 ng of purified DNA was used to construct reduced-representation libraries for sequencing. The libraries were subjected to high-throughput sequencing on an × 10 Sequencing System (Illumina) with a 150 bp paired-end sequencing strategy.

### SNP-calling

FastQC (http://www.bioinformatics.babraham.ac.uk/projects/fastqc/) and Fastp (v0.12.4) programs were employed for raw reads preprocessing (Chen et al. 2018 ). Subsequently, clean reads were aligned to the var. Chinese Spring reference genome (RefSeq v1.0) using BWA aln (v0.7.10-r789) (Li and Durbin 2009; Li et al. 2022). Samtools (v1.14) (Li et al. 2009) and the Genome Analysis Toolkit (GATK, v4.1.4.1) (McKenna et al. 2010) were utilized for processing SAM files and SNP-calling. Only high-quality SNPs were retained according to the following standards: (1) biallelic sites; (2) different SNPs between cv. Shi4185 and var. XJ5; (3) DP ≥ 2 for both cv. Shi4185 and var. XJ5; (4) missing rate within the IL population ≤ 0.5.

### Association analysis of grain weight per spike

Association analysis was performed using the mixed linear modeling (MLM) implemented in the genome-wide efficient mixed-model association (GEMMA) software (beta-07Mar2010 version) (Kang et al. 2010; Zhou and Stephens 2012). The grain weight per spike for each IL was analyzed. Since the Bonferroni correction method for multiple testing was too conservative and detected only a few significant SNPs, markers with adjusted −lg*P* ≥ 5 (control threshold) were considered significant for grain weight per spike (Zhang et al. 2015; Du et al. 2018).

Based on the number of SNPs analyzed (*n* = 101,895), the threshold *P*-value for genome-wide 5% and 1% significance were calculated as –lg(0.05/101,895) = 6.31 and –lg(0.01/101,895) = 7.01, respectively. Considering the complexity of the agronomic trait, SNPs that passed the control threshold score or had a −lg*P* score above the threshold were identified as significant positions associated with the target trait.

### RNA-seq and data analysis

For RNA-seq analysis, young spikes of cv. Shi4185, IL734 and IL915 at the glume differentiation stage and the immature grain at 6 d post anthesis were sampled. Approximate 1 mg of total RNA from each sample was used to construct a sequencing library, and all the libraries were sequenced on an Illumina HiSeq 2500 sequencer. The bioconductor package DESeq2 was used to determine DEGs with log_2_ (fold change) ≥ 1 and an adjusted *P* value < 0.05.

The syntenic genome regions of var. Chinese Spring (IWGSC V1.0) and cv. KN9204 (Shi et al. 2022) were analyzed with Mummer (v4.0.0rc1) using default parameters (Kurtz et al. 2004). The target sequence of var. Chinese Spring (Chr7A: 670.70∼675.30 mb) and the corresponding region of cv. KN9204 (Chr7A: 673.56∼678.35 mb) were extracted. Homoeologous genes between var. Chinese Spring and cv. KN9204 were identified using BLASTN (v2.11.0). Collinear segments were detected and displayed with JCVI software (v1.2.1, https://github.com/tanghaibao/jcvi).

### Constructs and plant transformation

A 3,562-bp genomic DNA fragment of the *CHLI-A* gene, including 1,744 bp of the 5′ upstream region, 1,442 bp of the *CHLI-A* gene coding sequence, and 376 bp of the 3′ downstream region, was PCR-amplified from IL915 genomic DNA with the *CHLI-A*_gOEF and *CHLI-A*_gOER primers, cloned into the pMOA36 vector to generate the pMOA36-*ProCHLI-A:CHLI-A* complementary construct. The construct was subsequently transformed into cv. Shi4185 via *Agrobacterium*-mediated gene transformation according to the previously described method (Wang et al. 2022a).

Using the flag leaf cDNA of IL915 as template, a 1266-bp coding region sequence of *CHLI-A* gene was amplified with *CHLI-A*_OEF/*CHLI-A*_OER primer set and cloned into p201 vector with *UBI* promoter to generate the p201-*ProUBI:CHLI-A* overexpression construct. For CRISPR/Cas9-based gene editing, a single-guide RNA (sgRNA) sequence was designed according to the exon sequences of wheat *CHLI-A* using CRISPR-Cereal (http://crispr.hzau.edu.cn/CRISPR-Cereal/index.php). The pCas9T vector was amplified using 2 pairs of primers containing the sgRNAs and then cloned and inserted into the CRISPR/Cas9 vector *pCas9T* (Pan et al. 2025). The overexpression and recombinant gene editing plasmids were subsequently transformed into cv. Fielder via *Agrobacterium*-mediated gene transformation respectively. All positive transgenic lines were identified using gene-specific primers. The primers used for DNA fragment amplification and construct design are listed in Supplementary Table S3.

### Transgene copy number quantification by real time PCR

To detect the *CHLI-A* transgene copy number in *ProCHLI-A:CHLI-A* transgenic wheat plants, genomic DNA was extracted using the standard CTAB method (Murray and Thompson 1980). The *TaSSII-D* gene served as a single-copy reference. Transgene copy numbers in *ProCHLI-A:CHLI-A* lines (cv. Shi4185 background) were quantified following a published protocol (Lee et al. 2008).

### Quantitative RT-PCR analysis

Total RNA was isolated from seedling leaves of cv. Shi4185, cv. Fielder, var. XJ5, transgenic plants, and mutants using the SPARKeasy New Plant RNA Rapid Extraction Kit (Cat. # AC0305, SparkJade Biotechnology) according to the manufacturer's instructions. First-strand cDNA synthesis were carried out using a reverse transcription kit (Cat. AU341-02, Transgen Biotechnology). RT-qPCR was carried out using SYBR Green PCR master mix (Cat. # DLQ104, Tsingke Biotechnology). Each assay included at least 3 biological replicates with 3 technical replicates. The wheat *ACTIN* gene (*TraesCS1D02G274400*) served as an internal reference. Primer sequences are listed in Supplementary Table S3.

### Chlorophyll content measurement and photosynthetic rate detection

The chlorophyll content and photosynthetic rate were measured at flowering stage in the field. The chlorophyll content was converted and calculated using the SPAD values measured by SPAD502 (Ling et al. 2011). The SPAD value for each leaf is expressed as the average of 5 SPAD values from 5 random points on the flag leaf of each plant, and the final SPAD value for each line were the average of at least 5 independent plants. The photosynthetic rate was measured using an LI-6800 instrument, and the final values for each line were the average of at least 10 independent plants.

### Phylogenetic analysis

Protein sequences of CHL subunits from various species were obtained from the National Center for Biotechnology Information (https://www.ncbi.nlm.nih.gov/). Multiple sequence alignment was performed using Clustal Omega software (https://www.ebi.ac.uk/jdispatcher/msa/clustalo), and a phylogenetic tree was constructed using the neighbor-joining method in MEGA 6 software with default parameters. Evolutionary distances were calculated using the Poisson model, and the phylogenetic test was conducted using the bootstrap method with 500 replications. The protein sequences and sequence alignment files are provided in Supplementary Files 1 and 2.

### Statistics and data visualization

Quantitative data are presented as mean ± standard deviation. One-way analysis of variance (ANOVA) and Dunnett's multiple comparison test were conducted for comparing the difference of RPKM values and thousand-grain weights among the wheat accessions corresponding to 6 *CHLI-A* haplotypes, respectively, with different letters indicating significant differences at *P* < 0.05 and the same letters indicating nonsignificances. Comparisons between 2 sample means were performed using Student's two-tailed *t-*tests. Significant differences are indicated by one star (*) for at *P* < 0.05 and 2 stars (**) for *P* < 0.01 in corresponding figures. Data were visualized using GraphPad Prism 8 and Excel 2019 software. Detailed statistical analysis data are present in Supplementary Data Set 9.

### Accession numbers

Sequence data from this article can be found in the EnsemblPlants database (http://plants.ensembl.org/index.html) under the following accession numbers: *CHLI-A* (*TraesCS7A02G480700*), *CHLI-B* (*TraesCS7B02G382800*), *CHLI-D* (*TraesCS7D02G467500*), *TaLHCB1-A* (*TraesCS7A02G276400*), *TaLHCB1-D* (*TraesCS7D02G276300*), *TaPEPC-A* (*TraesCS6A02G195600*), *TaPEPC-D* (*TraesCS6D02G183200*), *TaRBCS-A* (*TraesCS2A02G067100*), *TaRBCS-B* (*TraesCS2B02G078900*), and *TaRBCS-D* (*TraesCS2D02G065500*). The raw sequence data reported in this paper have been deposited in the Genome Sequence Archive at the National Genomics Data Center, China National Center for Bioinformation / Beijing Institute of Genomics, Chinese Academy of Sciences, under accession number CRA017446. These data are publicly accessible at https://ngdc.cncb.ac.cn/gsa.

## Supplementary Material

koaf212_Supplementary_Data

## Data Availability

The data for this research are available in the article and in the online Supplementary material and dataset.

## Supplementary data

The following materials are available in the online version of this article.

**Supplementary Figure S1.** Validation of *QGwps.pku-7A*.

**Supplementary Figure S2.** Assessing the effects of *QGwps.pku-7A* using ILs from 2019 and 2020.

**Supplementary Figure S3.** The transcript expression of 3 homoeologous *CHLI* genes.

**Supplementary Figure S4.** Rescuing *CHLI-A* in cv. Shi4185 does not change plant height, spike number per plant and spike-related traits.

**Supplementary Figure S5.**  *CHLI-A* overexpression does not change plant height, spike number per plant and spike-related traits in cv. Fielder.

**Supplementary Figure S6.** Comparison of leaf phenotypes and chlorophyll contents at the seedling stage.

**Supplementary Figure S7.**  *CHLI-A* increases chlorophyll *a* and *b* contents.

**Supplementary Figure S8.**  *CHLI-A* increases the sizes of glumes, lemmas and paleas in cv. Shi4185.

**Supplementary Figure S9.** Expression levels of genes encoding photosynthetic pathway proteins in cv. Shi4185 and *ProCHLI-A:CHLI-A* transgenic lines.

**Supplementary Figure S10.** Expression levels of genes encoding photosynthetic pathway proteins in cv. Fielder, *chli-A*, and *ProUBI:CHLI-A* transgenic lines.

**Supplementary Figure S11.** Field evaluation of the yield components at the population level.

**Supplementary Figure S12.**  *CHLI-A* PAV distribution in Chinese wheat-producing provinces.

**Supplementary Figure S13.** Phylogenetic analyses of CHLI homologs in dicots and monocots.

**Supplementary Table S1.** Quantification of the high-quality SNPs used for the association analysis in this study.

**Supplementary Table S2.** Copy number variation of the *CHLI-A* gene in *ProCHLI-A:CHLI-A* transgenic lines.

**Supplementary Table S3.** Primers used in this study.

**Supplementary Data Set 1.** Fragment introgression from var. XJ5 into cv. Shi4185.

**Supplementary Data Set 2.** Significant SNPs and genes located at the *QGwps.pku-7A* locus.

**Supplementary Data Set 3.** DNA polymorphism between cv. Shi4185 and var. XJ5 at the *QGwps.pku-7A* locus.

**Supplementary Data Set 4.** DNA polymorphisms in the coding region of the genes between cv. Shi4185 and var. XJ5 at the *QGwps.pku-7A* locus.

**Supplementary Data Set 5.** FPKM values for the genes at the *QGwps.pku-7A* locus among IL734, IL915 and cv. Shi4185.

**Supplementary Data Set 6.** Expression profile of the genes at the *QGwps.pku-7A* locus in different tissues at various wheat developmental stages.

**Supplementary Data Set 7.** FPKM values for the differently expressed genes (DEGs) among IL734, IL915 and cv. Shi4185.

**Supplementary Data Set 8.** Detection of the cross-population composite likelihood ratio (XP-CLR) of *CHL* genes.

**Supplementary Data Set 9.** Results of the statistical analyses in this study.

**Supplementary File 1.** Protein sequences used to generate the phylogenetic tree shown in Supplementary Fig. S12.

**Supplementary File 2.** Protein sequence alignment used to generate the phylogenetic tree shown in Supplementary Fig. S12.

## Dive Curated Terms

The following phenotypic, genotypic, and functional terms are of significance to the work described in this paper:

CHLD Gramene: AT1G08520
CHLD Araport: AT1G08520

CHLH Gramene: AT5G13630
CHLH Araport: AT5G13630

chlorophyll CHEBI: CHEBI:28966

chlorophyll content Planteome: TO:0000495

HAE Gramene: AT4G28490
HAE Araport: AT4G28490
