# Supplementary material for: Natural variation in CHELATASE SUBUNIT I-A increases grain weight and enhances wheat yield
Source: Plant Cell. 2025 Aug 30;37(9):koaf212. doi: 10.1093/plcell/koaf212 (PMC12419692; doi:10.1093/plcell/koaf212)
Supplement: koaf212_Supplementary_Data [file koaf212_supplementary_data.zip › Supplemental material-PDF.pdf]

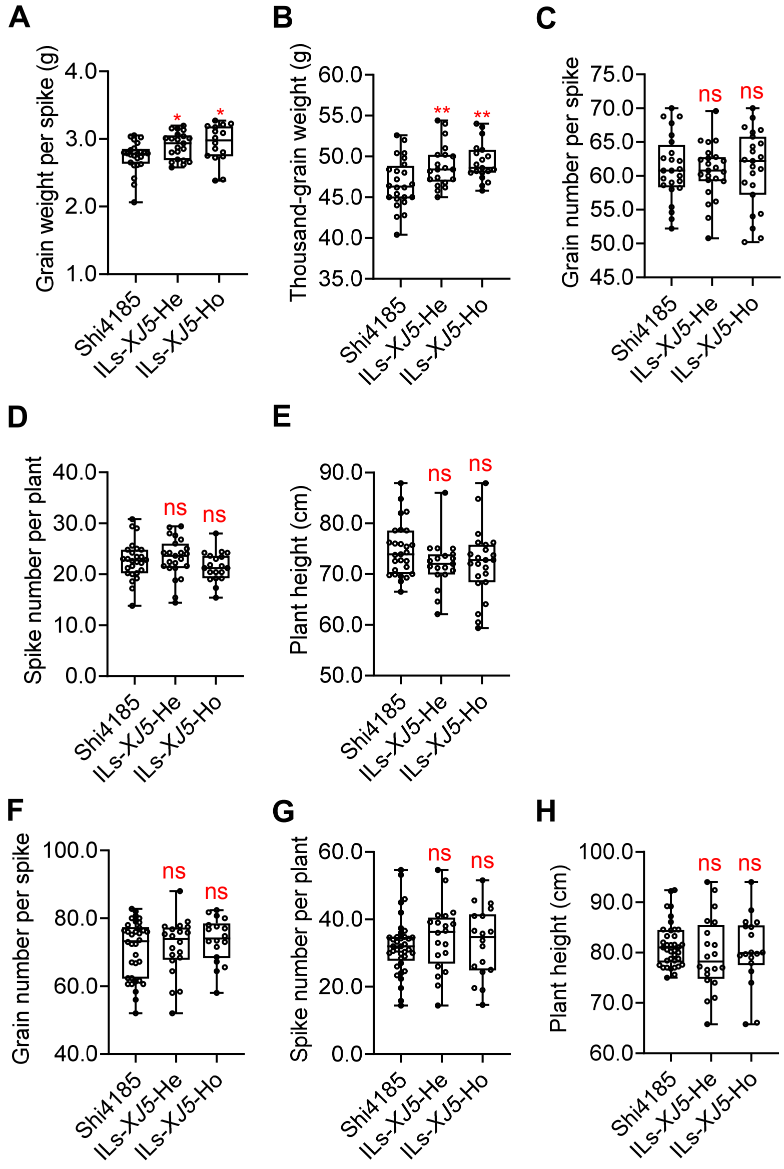

Supplementary Figure S1. Validation of *QGwps.pku-7A*. (Supports Figure 1) **A** to **E)** Comparative analysis of grain weight per spike (**A**), thousand-grain weight (**B**), grain number per spike (**C**), spike number per plant (**D**) and plant height (**E**) among pooled ILs with homozygous (Ho) or heterozygous (He) introgressed *QGwps.pku-7A* and cv. Shi4185, respectively, according to the agronomic data measured in field in 2020. **F** to **H)** Comparative analysis of grain number per spike (**F**), spike number per plant (**G**) and plant height (**H**) among pooled ILs with homozygous (Ho) or heterozygous (He) introgressed *QGwps.pku-7A* and cv. Shi4185 respectively, according to the agronomic data measured in field in 2019. Boxes represent interquartile range, horizontal lines

denote median, whiskers = min - max, n = 18 to 25. ns, nonsignificance. *, $P < 0.05$; **, $P < 0.01$ indicate significant differences compared with cv. Shi4185 for all statistical analyses (two-tailed Student's *t*-test).

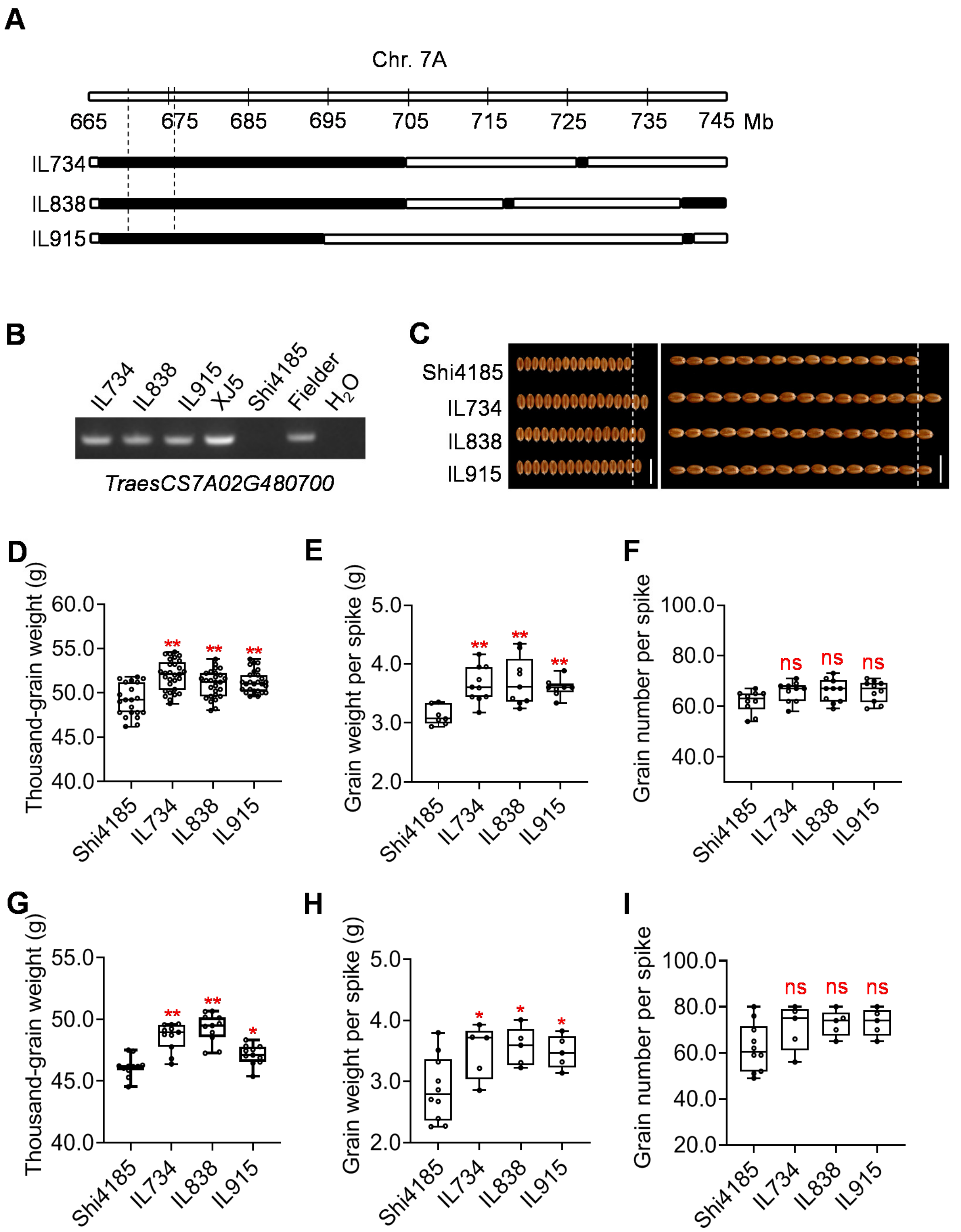

Supplementary Figure S2. Assessing of the effects of *QGwps.pku-7A* using ILs from 2019 and 2020. (Supports Figure 1 and 2) **A)** Illustration of the introgressed exogenic XJ5 chromosome fragments (black regions) on the cv. Shi4185 background (white) in the IL734, IL838 and IL915. The white dash lines confined the *QGwps.pku-7A* region. **B)** Detection of the candidate gene *TraesCS7A02G480700* in ILs, var. XJ5, cv. Shi4185, and cv. Fielder. **C)** Comparisons of grain width and grain length between cv. Shi4185 and each IL. Scale bar, 1 cm. **D** and **F)** Comparative analysis of the thousand-grain weight

(**D**), grain weight per spike (**E**) and grain number per spike (**F**) between cv. Shi4185 and each IL in fielder in 2019. **G** and **I)** Comparative analysis of the thousand-grain weight (**G**), grain weight per spike (**H**) and grain number per spike (**I**) between cv. Shi4185 and each IL in fielder in 2020. Boxes represent interquartile range, horizontal lines denote median, whiskers = min - max, n = 5. *, $P < 0.05$; **, $P < 0.01$, significant differences compared with cv. Shi4185 for all statistical analyses (two-tailed Student's *t*-test). ns indicates nonsignificance (two-tailed Student's *t*-test).

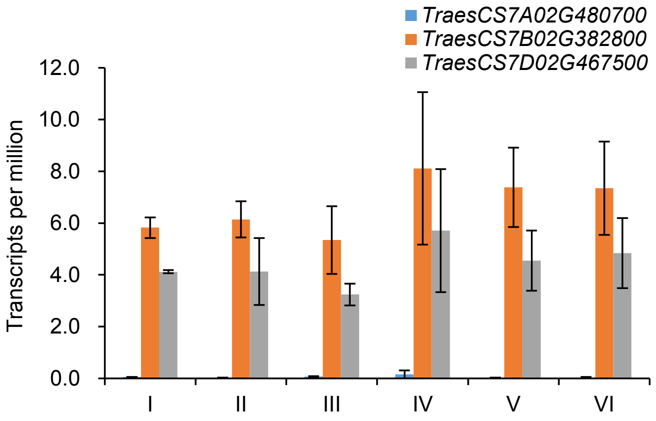

Supplementary Figure S3. The transcript expression of three homoeologous *CHLI* genes. (Supports Figure 2) Expression of *TraesCS7A02G480700*, *TraesCS7B02G382800* and *TraesCS7D02G467500*, at different young spike development stages in cv. KN9204. Stages I-VI indicated the vegetative stage, elongation stage, single ridge stage, double ridge stage, glume primordium differentiation stage, and floret differentiation stage. The data are shown as mean ± SD (n = 3).

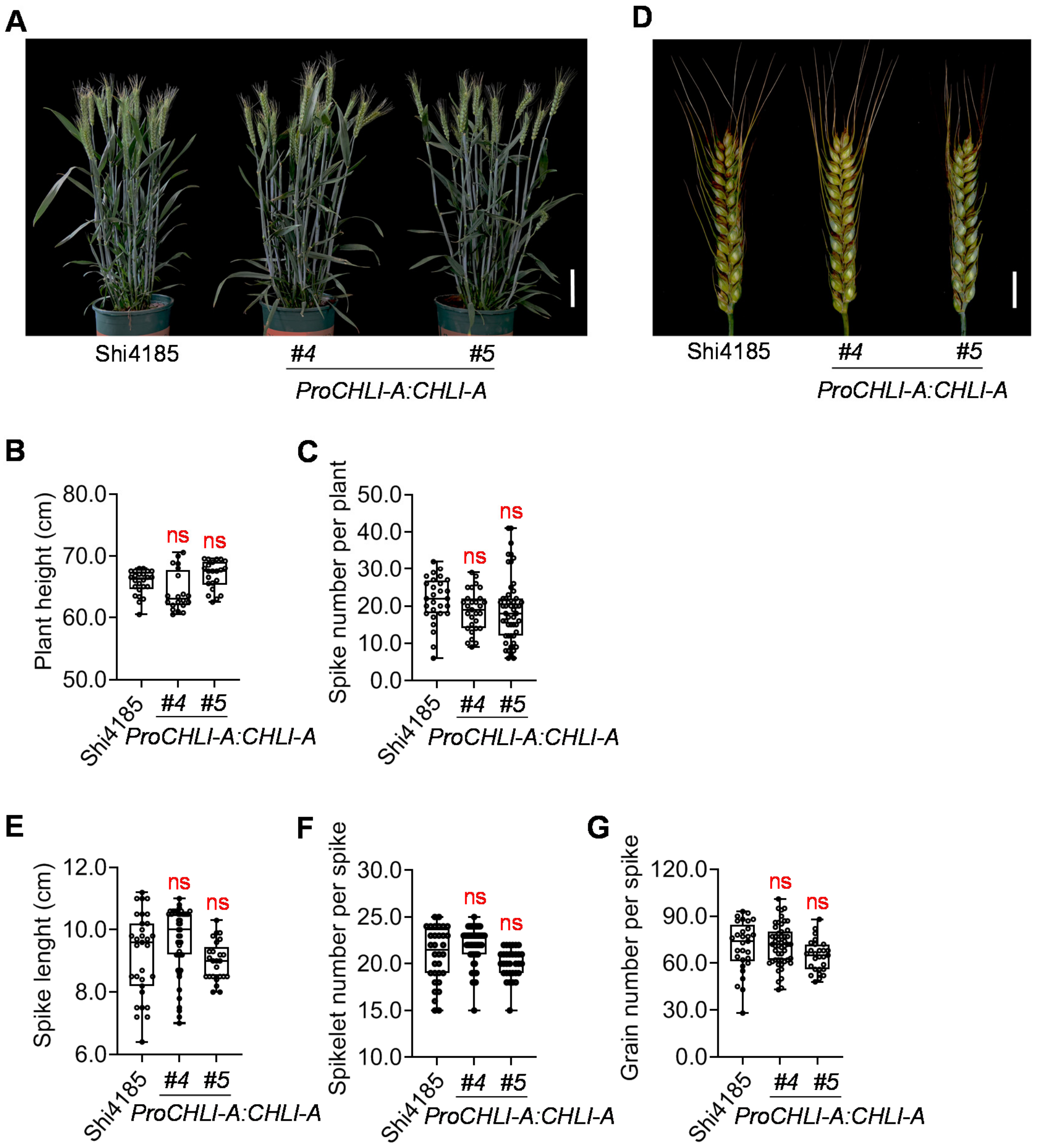

Supplementary Figure S4. Rescuing *CHLI-A* in cv. Shi4185 did not change plant height, spike number per plant and spike-related traits. (Supports Figure 3) **A)** Phenotypic comparison of plant height among cv. Shi4185 and *ProCHLI-A:CHLI-A* transgenic lines. Images were digitally extracted for comparison. Scale bar, 10 cm. **B** and **C)** Comparative analysis of the plant height (**B**) and spike number per plant (**C**) between cv. Shi4185 and *ProCHLI-A:CHLI-A* transgenic lines. **D)** Phenotypic comparison of spike structure between cv. Shi4185 and each *ProCHLI-A:CHLI-A* transgenic line. Images were digitally extracted for comparison. Scale bar, 2 cm. **E** to **G)** Comparison of spike length (**E**), spikelet number per spike (**F**)**,** and grain number per spike (**G**) between cv.

Shi4185 and each *ProCHLI-A:CHLI-A* transgenic line. Boxes represent interquartile range, horizontal lines denote median, whiskers = min - max, n = 20 to 50. ns, nonsignificance. *, P < 0.05; **, P < 0.01 indicate significant differences compared with cv. Shi4185 for all statistical analyses (two-tailed Student's *t*-test). All compared plants were grown in the field.

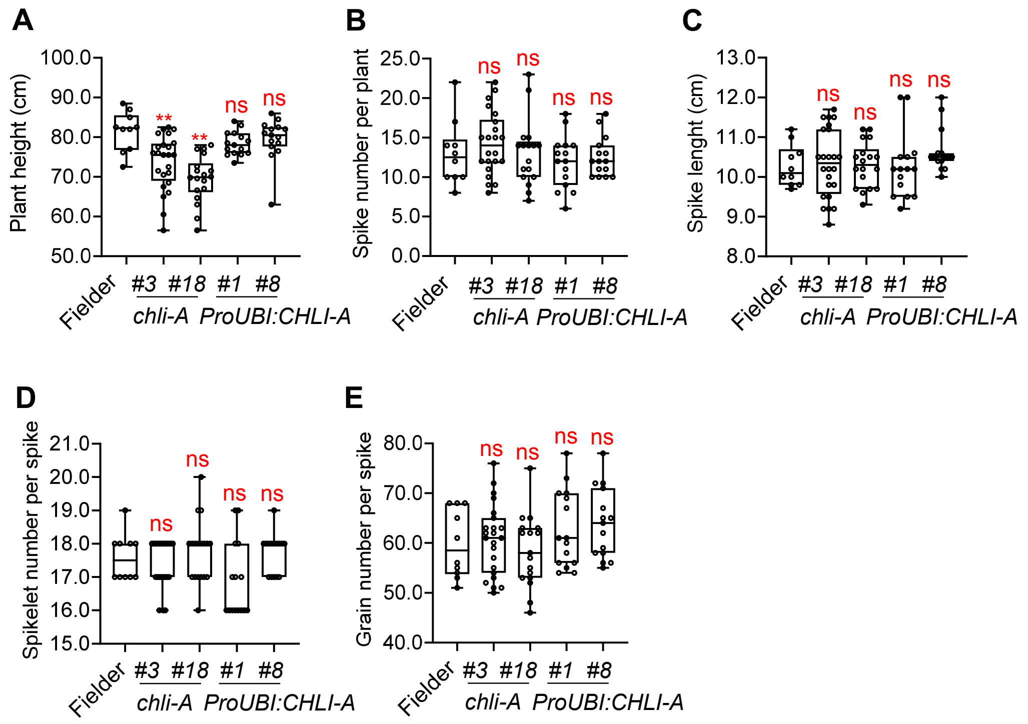

Supplementary Figure S5. *CHLI-A* overexpression does not change plant height, spike number per plant, or spike-related traits in cv. Fielder. (Supports Figure 4) **A** to **E)** Comparative analysis of the plant height (**A**), spike number per plant (**B**), spike length (**C**), spikelet number per spike (**D**), and grain number per spike (**E**) between cv. Fielder and *chli-A* mutants or *ProUBI:CHLI-A* transgenic lines grown in the field. Boxes represent interquartile range, horizontal lines denote median, whiskers = min - max, n = 10 to 24. ns indicates nonsignificance. **, $P < 0.01$ indicate significant differences compared with cv. Fielder for all statistical analyses (two-tailed Student's t-test).

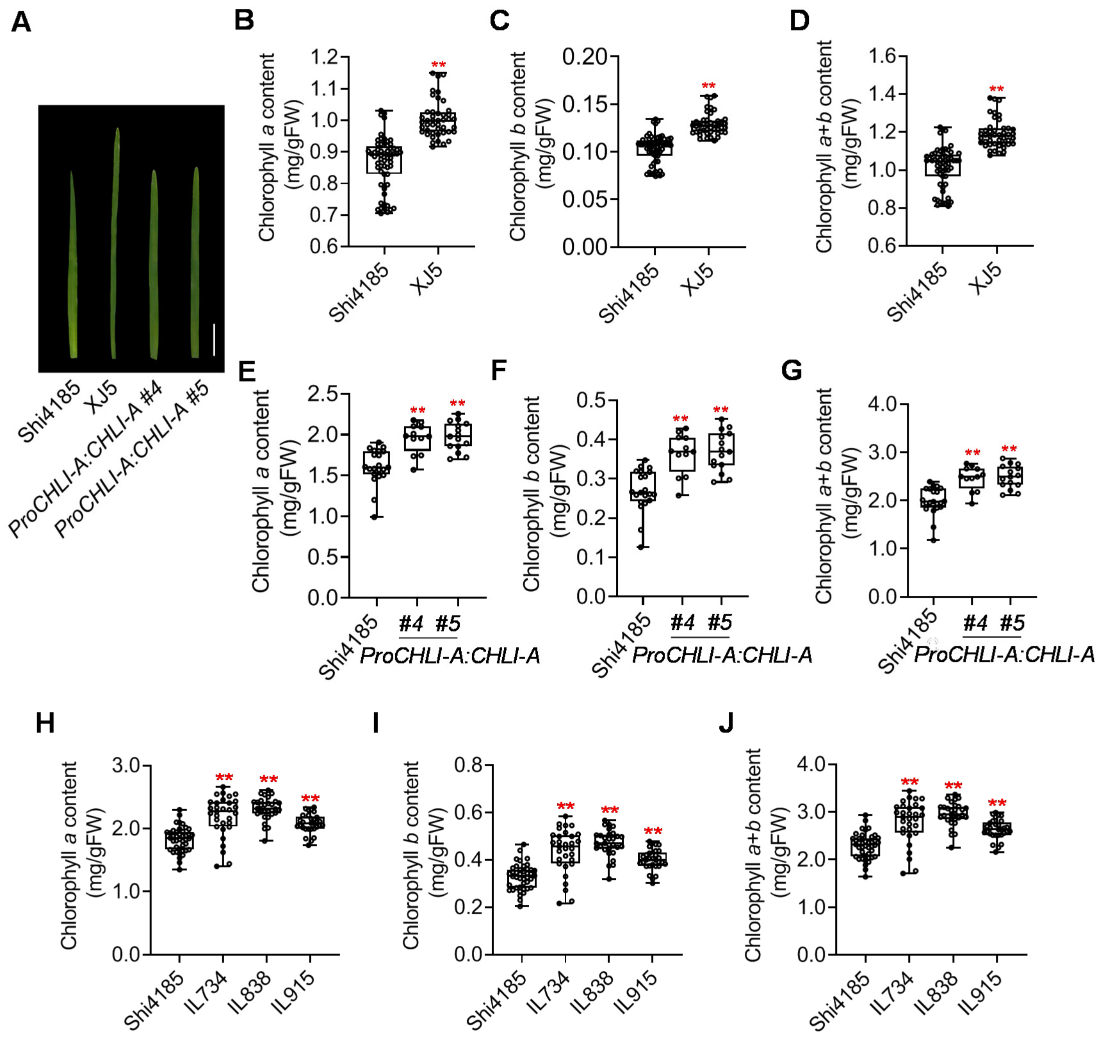

Supplementary Figure S6. Comparison of the leaf phenotypes and chlorophyll contents at the seedling stage. (Supports Figure 5) **A)** Phenotypic comparison of seedlings between cv. Shi4185, var. XJ5, and *ProCHLI-A:CHLI-A* lines. Images were digitally extracted for comparison. Scale bar, 2 cm. **B** to **J**) Comparisons of chlorophyll contents between cv. Shi4185 and var. XJ5 (n = 44 to 60) (**B** to **D**), between cv. Shi4185 and *ProCHLI-A:CHLI-A* transgenic lines (n = 12 to 21) (**E** to **G**), and between cv. Shi4185 and ILs with the *QGwps.pku-7A* (n = 30 to 42) (**H** to **J**). Boxes represent interquartile range, horizontal lines denote median, whiskers = min - max, n = 10 to 24. **, $P < 0.01$ indicates significant differences compared with cv. Shi4185 in all statistical analyses (two-tailed Student's *t*-test).

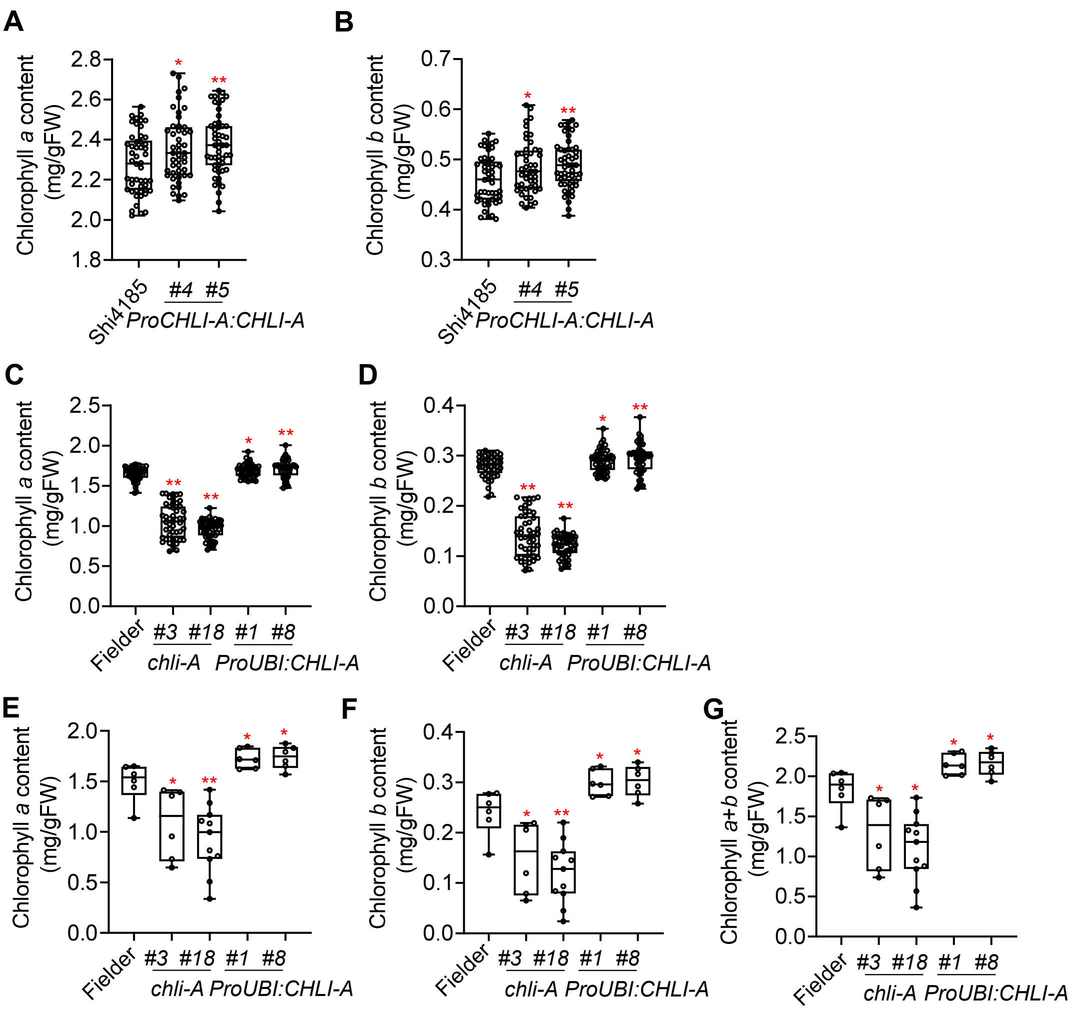

Supplementary Figure S7. *CHLI-A* increases chlorophyll *a* and *b* contents. (Supports Figure 5) **A** and **B)** Comparisons of the contents of chlorophyll *a* (**A**) and chlorophyll *b* (**B**) in flag leaves at the flowering stage between cv. Shi4185 and *ProCHLI-A:CHLI-A* transgenic lines, respectively. Boxes represent interquartile range, horizontal lines denote median, whiskers = min - max, n = 46 to 51. *, $P < 0.05$; **, $P < 0.01$ indicate significant differences compared with cv. Shi4185 in all statistical analyses (two-tailed Student's *t*-test). **C** to **G)** Comparative analysis of the contents of chlorophyll *a* (**C**) and chlorophyll *b* (**D**) in flag leaves at the flowering stage (n = 45 to 49), and chlorophyll *a* (**E**), chlorophyll *b* (**F**) and total chlorophyll (**G**) in leaves at the seedling stage (n = 6 to 11) between cv. Fielder and *chli-A* mutants, or cv. Fielder and *ProUBI:CHLI-A* transgenic lines, respectively. *, $P < 0.05$; **, $P < 0.01$ indicate significant differences compared with cv. Fielder in **(C)** to **(G)** (two-tailed Student's *t*-test).

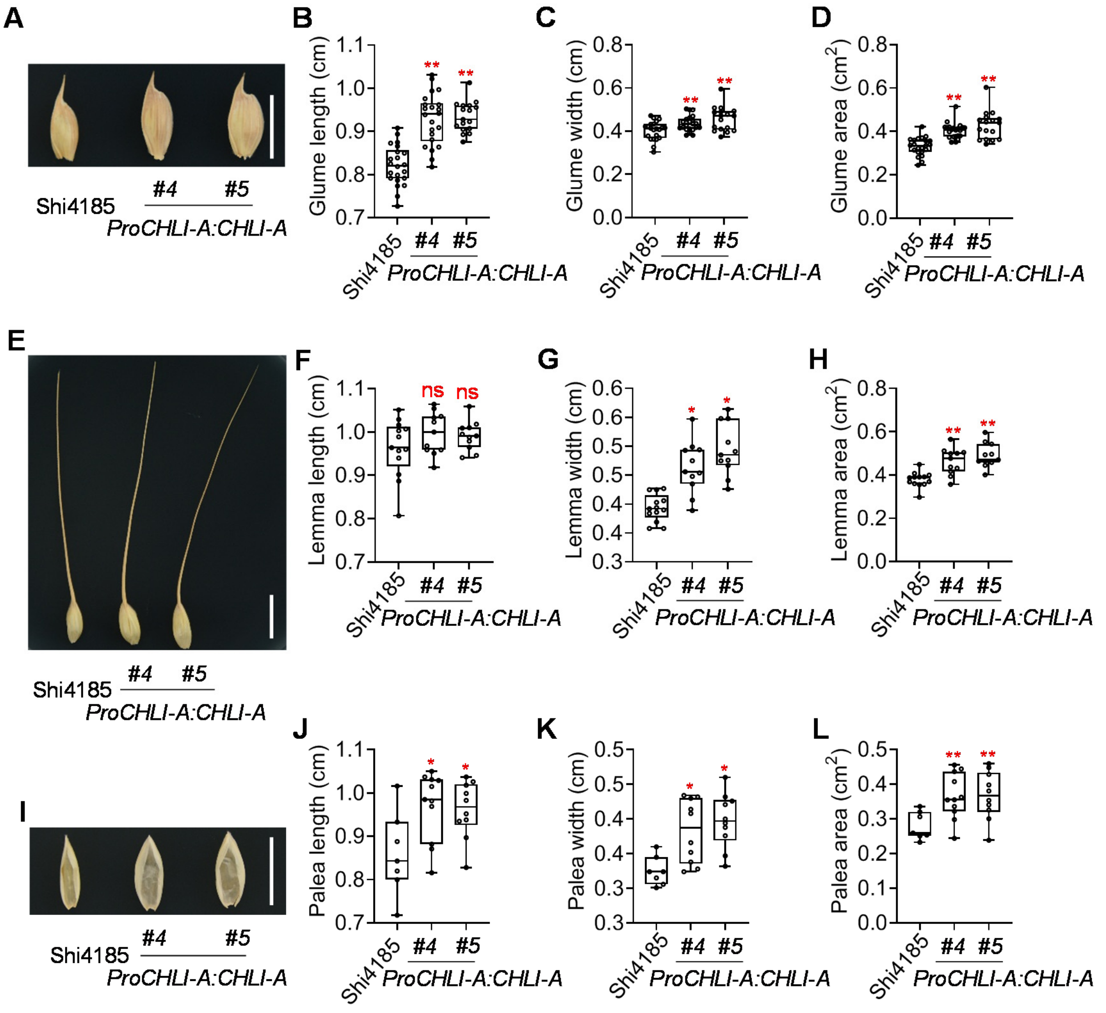

Supplementary Figure S8. *CHLI-A* increases the sizes of glumes, lemmas and paleas in cv. Shi4185. (Supports Figure 5) **A)** The *ProCHLI-A:CHLI-A* transgenic lines had bigger glumes than those of cv. Shi4185. Scale bar, 1 cm. **B** to **D)** Comparisons of glume length (**B**), width (**C**) and size (**D**) between cv. Shi4185 and two independent *ProCHLI-A:CHLI-A* transgenic lines (n = 17 to 22). **E)** The *ProCHLI-A:CHLI-A* transgenic lines had bigger lemmas than those of cv. Shi4185. Scale bar, 1 cm. **F** to **H)** Comparisons of the lemma length (**F**), width (**G**), and size (**H**) between cv. Shi4185 and *ProCHLI-A:CHLI-A* transgenic lines (n = 11 to 13). **I)** The *ProCHLI-A:CHLI-A* transgenic lines had bigger palea than those of cv. Shi4185. Scale bar, 1 cm. **J** to **L)** Comparisons of the palea length (**J**), width (**K**), and size (**L**) between cv. Shi4185 and *ProCHLI-A:CHLI-A* transgenic lines (n = 7 to 11). Boxes represent interquartile range, horizontal

lines denote median, whiskers = min – max. ns indicates nonsignificance. *, $P < 0.05$; **, $P < 0.01$ indicate significant differences compared with cv. Shi4185 in all statistical analyses (two-tailed Student's *t*-test).

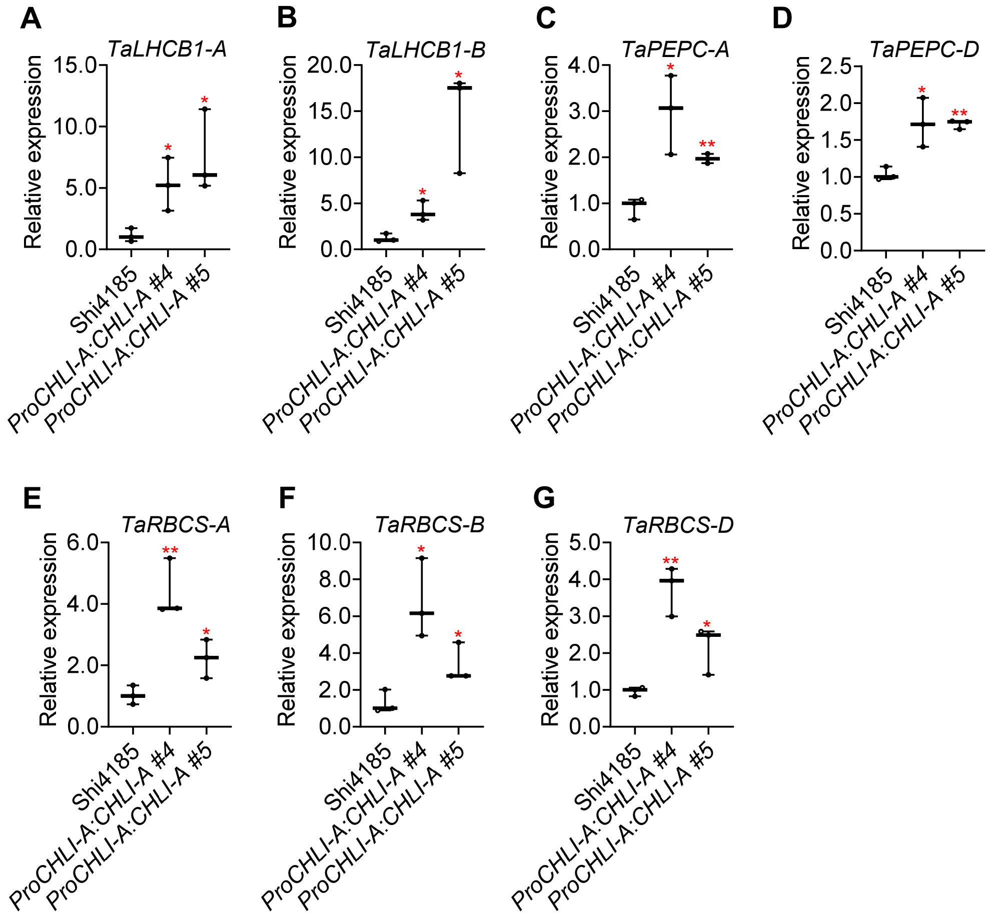

Supplementary Figure S9. Expression levels of genes encoding photosynthetic pathway proteins in cv. Shi4185 and *ProCHLI-A:CHLI-A* transgenic lines. (Supports Figure 5) Data were shown as mean ± SD of three biological replicates obtained by reverse transcription-quantitative PCR. *, $P < 0.05$; **, $P < 0.01$ indicate significant differences compared with cv. Shi4185 in all statistical analyses (two-tailed Student's *t*-test).

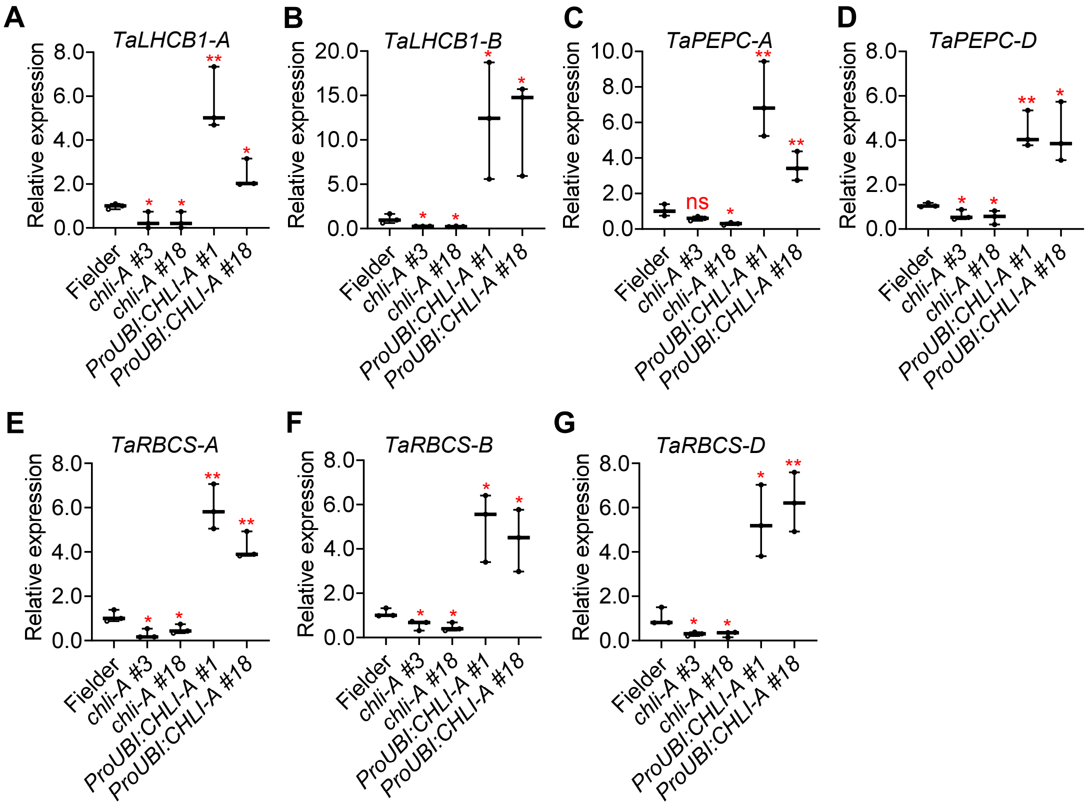

Supplementary Figure S10. Expression levels of genes encoding photosynthetic pathway proteins in cv. Fielder, *chli-A*, and *ProUBI:CHLI-A* transgenic lines**.** (Supports Figure 5) Data were shown as mean ± SD of three biological replicates obtained by reverse transcription-quantitative PCR. ns indicates nonsignificance. *, $P < 0.05$; **, $P < 0.01$ indicate significant differences compared with cv. Fielder in all statistical analyses (two-tailed Student's *t*-test).

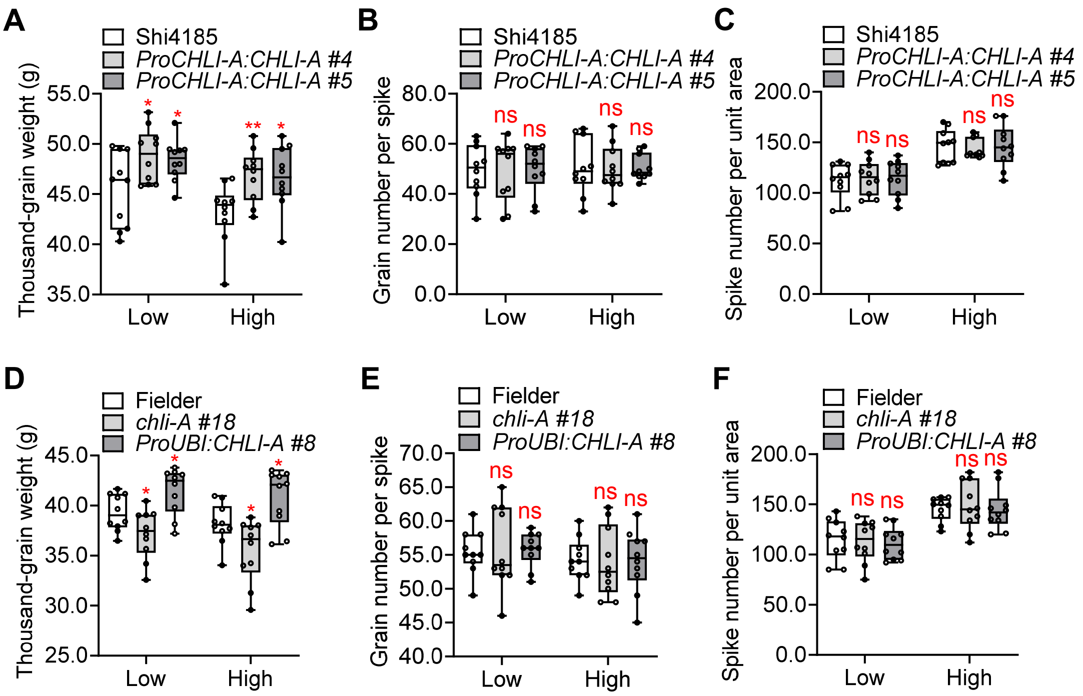

Supplementary Figure S11. Field evaluation of the yield components at the population level. (Supports Figure 6) Data for *CHLI-A* rescuing transgenic lines in cv. Shi4185 **A** to **C)**, the *CHLI-A* gene editing and overexpression lines in cv. Fielder **D** to **F)** are shown. Boxes represent interquartile range, horizontal lines denote median, whiskers = min - max, n = 10. ns indicates nonsignificance. *, P < 0.05; **, P < 0.01 indicate significant differences compared with related controls in all statistical analyses (two-tailed Student's t-test).

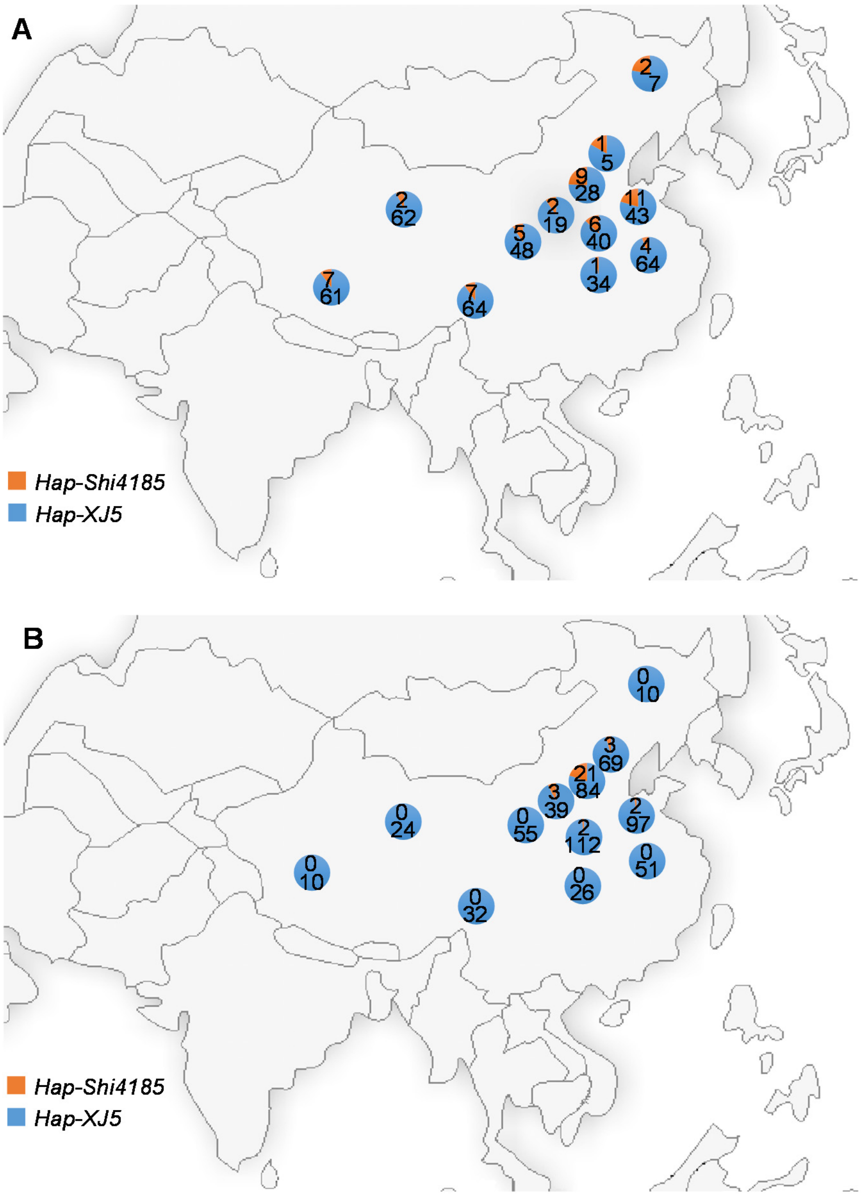

Supplementary Figure S12. *CHLI-A* PAV distribution across Chinese wheat-producing provinces (Supports Figure 6). The upper pie chart (**A**) illustrates landrace cultivars, while the lower pie chart (**B**) represents elite cultivars. The numerical values denote the total number of varieties in each category.

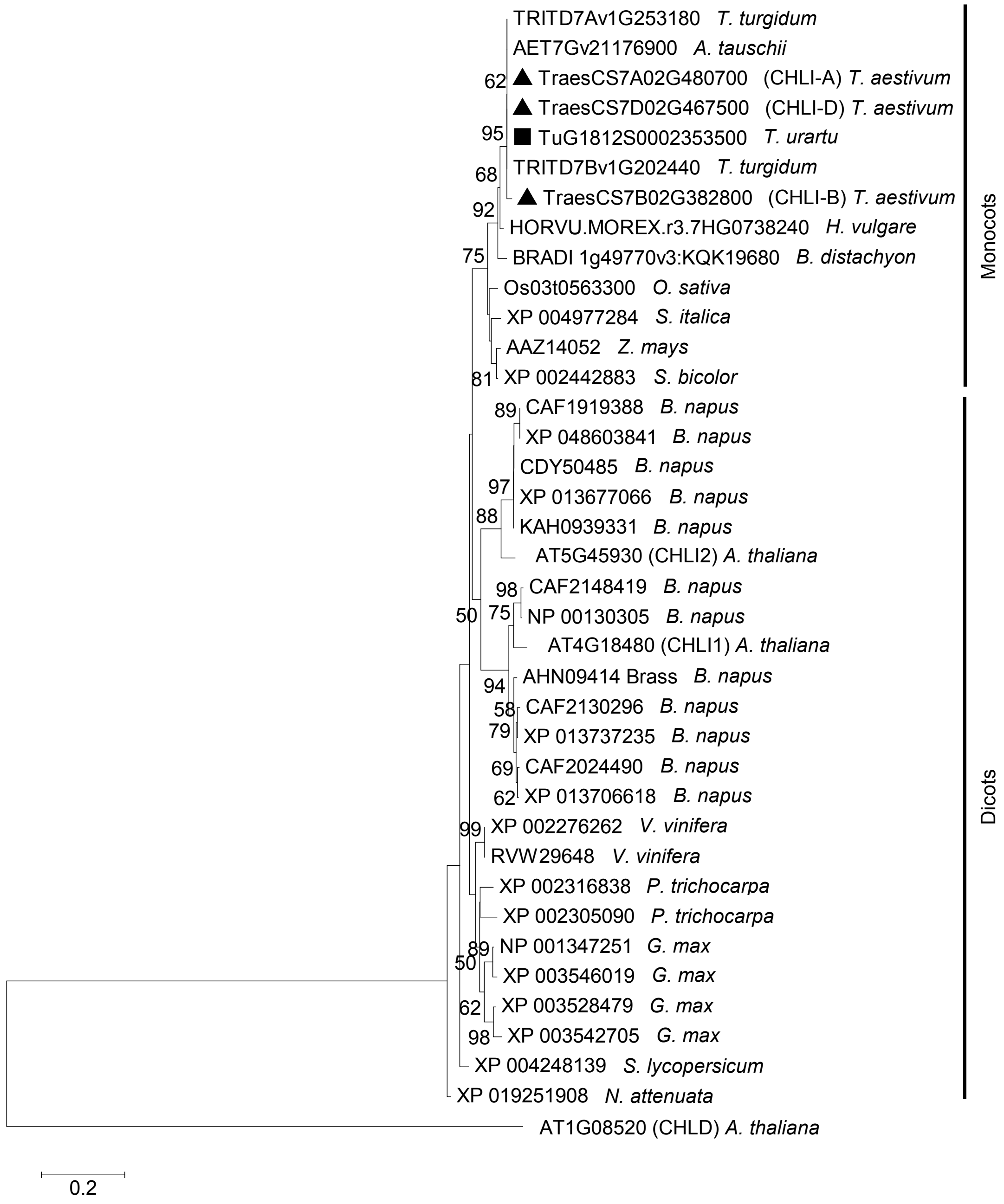

Supplementary Figure S13. Phylogenetic analyses of CHLI homologs in dicots and monocots. (Supports Figure 6) A neighbor-joining tree was generated by using MEGA6 and the numbers at each node show bootstrap values obtained for 500 replicates. The scale bar represents 0.2 amino acid substitutions per site. *Arabidopsis* CHLD was used as the outgroup. Solid triangles indicate three homeologous CHLIs in common wheat, and the solid square is the CHLI in *T. urartu*.
