# Supplementary material for: Natural variation in CHELATASE SUBUNIT I-A increases grain weight and enhances wheat yield
Source: Plant Cell. 2025 Aug 30;37(9):koaf212. doi: 10.1093/plcell/koaf212 (PMC12419692; doi:10.1093/plcell/koaf212)
Supplement: koaf212_Supplementary_Data [file TPC-2025-0116R1 peer review report.pdf › TPC-2025-0116R1 peer review report.pdf]

Date: Jul 16, 2025
To: "qiang gao" qianggao@cau.edu.cn
cc: "Ying Zang" zb20193020217@cau.edu.cn, "Ji-Hui Qiao" sz20183020170@cau.edu.cn, "De-Shui Liu" deshui@yeah.net, "Dong-Min Gao" 85012885@qq.com, "Xin-Wen Zhang" sz20213020221@cau.edu.cn, "Tian-Tian Pan" zb20243020259@cau.edu.cn, "Yi-Zhou Yang" yizhou.yang@qq.com, "Xian-Bing Wang" wangxianbing@cau.edu.cn
From: "The Plant Cell" tpc-submissions@aspb.org
Subject: TPC-2025-0116R1 Decision Letter

Ref.: Ms. No. TPC-2025-0116R1
Barley m6A demethylase HvALKBH1B undergoes phase separation to enhance antiviral immunity against a plant rhabdovirus
The Plant Cell

Dear gao,

I am pleased to inform you that your revised manuscript "Barley m6A demethylase HvALKBH1B undergoes phase separation to enhance antiviral immunity against a plant rhabdovirus" has been accepted for publication in The Plant Cell.

At this stage, your manuscript will be sent for additional pre-publication science editing if it is one of the following article types: Research Article, Large-Scale Biology, Breakthrough Report. The Science Editors will review the manuscript with respect to its presentation of scientific content, compliance with journal policies, and presentation for a broad readership. The Plant Cell has contracted with Plant Editors (planteditors.com) to provide this service to our authors, and you will soon receive additional information on this process.

ORCID ID NEEDED: Please note that each author needs to link their ORCID identifier to their account in the system before your manuscript can be published. If any authors do not have an ORCID linked to their account, they will receive a message with a link to complete this task. Please ensure that ALL of your coauthors have completed this task as soon as possible.

LICENSE INFORMATION Shortly after your paper is sent to production, you will receive an email from our publisher's author support team, SciPris, with information on article processing charges, as well as the types of Open Access license you would be able to purchase. The accepted version of your manuscript will not appear on our Advance Articles page until the license is selected and signed, so please look out for an email from SciPris. For more information see the Article Publication Fees section of our author guidelines: https://academic.oup.com/plcell/pages/General-Instructions

The Plant Cell and The Arabidopsis Information Resource (TAIR) are collaborating to collect functional annotation data about Arabidopsis genes from authors. This includes information about the gene's molecular function (e.g., kinase activity, ATP synthetase activity), the biological process/es it is involved in (e.g., endosperm development, threonine biosynthesis), its subcellular location (e.g., nucleus, ER), anatomical or developmental expression pattern (e.g., leaf, ovule, flower stage 10, seedling stage), or its partner in a protein-protein interaction (e.g., AT1G01010 interacts with AT1G01020).

If your paper contains results falling into one or more of these categories for Arabidopsis genes, we request that you now submit these data for inclusion in TAIR by filling in the form provided at the following URL: https://www.arabidopsis.org/doc/submit/functional_annotation/123. If you need further clarification on what types of data can be submitted please contact curator@arabidopsis.org.

Finally, we encourage your submission of artwork for the journal cover. If there is an image or illustration related to your paper that you would like to have considered, please email it in .pdf or .tif format to clowe@aspb.org. An image title, brief, 2-3 sentence description of the image, and the name of the person credited for the image are also encouraged. If your image is selected to appear on the online cover of the journal, you will be contacted with the scheduled issue date and further information.

We look forward to seeing your paper published.

On behalf of the editorial board,

Sincerely,

Sara Lopez-Gomollon, PhD, Guest Editor

Pablo Manavella, Editor-in-Chief
The Plant Cell

=====
IMPORTANT REMINDER: PEER REVIEW REPORTS
=====

If you opted to publish a peer review report along with your article during the original submission process, it will be prepared by the editorial staff and publicly posted with your manuscript, inside the zip file that contains any other supplemental material. As a reminder, the peer review report is a public record of all comments from editors and reviewers, as well as your prior responses, as you received them in the decision letters for each draft of your manuscript. If you agreed to publish this report and have changed your mind, or are not sure if you selected this option, please contact the editorial office as soon as possible before signing the license agreement from our publisher.

Comments from the Editors and Reviewers:

Reviewers' comments:
Reviewer 1: In this new version, the authors have addressed most, if not all, of the concerns raised in the first version by performing the necessary experiments. They have demonstrated the specificity of the m6A antibody, quantitatively analyzed fluorescence, dissected the minimal motifs and/or amino acids required for phase separation, and ruled out other demethylases as possible players in the antiviral activity. Furthermore, they have now convincingly explained why they did not use a full-length version of the demethylase in the overexpression experiments. Therefore, as far as I am concerned, the authors have successfully justified all my concerns.

Reviewer 2: This revised version has addressed my concerns.

****

Ref.: Ms. No. TPC-2025-0116R1
Barley m6A demethylase HvALKBH1B undergoes phase separation to enhance antiviral immunity against a plant rhabdovirus
The Plant Cell

In compliance with data protection regulations, you may request that we remove your personal registration details at any time. (Use the following URL: https://www.editorialmanager.com/tpc/login.asp?a=r). Please contact the publication office if you have any questions.

Firstly, we would like to thank the editors and reviewers for all their efforts in assessing our manuscript and providing helpful and constructive suggestions. We have addressed these comments through performing additional experiments and modifying statements. The manuscript is indeed much improved. Comments by the editors and reviewers are shown in black, and our point-by-point response is shown in blue.

A note from the Editor-in-Chief: We are trying to make a concerted effort to change green/red comparisons to green/magenta to make our figures understandable to those with color vision deficiencies. We noticed that a small number of your figures (for example Fig. 2A, 4K, 5E) utilize red/green contrasts, so as you prepare the final version of the figures, please check the figures for red/green color use. Magenta is a good substitute for red, and a light blue substitutes for green even if the data are labeled as "RFP" or "mCherry" - readers will understand that colors can be changed, and indeed, it can be adjusted relatively quickly by using the "hue" setting. You might want to check over the Supplemental Figures for similar comparisons. Please note that color changes do not need to be highlighted or tracked in the revised manuscript, but could be noted in the cover letter or response document.

Given the nature of the comments, we are offering you 60 days from when we have issued this decision to complete the revision. If a revision is not returned within this time frame, and if you have not been granted an extension, we will withdraw the manuscript, which will leave you free to submit the work elsewhere. If you need an extension, we encourage you to contact us at any point before the 60 days have passed.

When you are ready to submit the revised version, please upload a highlighted copy that indicates all changes made in response to the editor and reviewer recommendations. Include an itemized list of all changes made in response to each of the reviewer's suggestions in the "Response to Reviewers" section; please note that reviewers do not have access to your cover letter, nor was this decision letter shared with them.

Thank you for the privilege of reviewing your work. We look forward to receiving your revised manuscript.

Yours sincerely

Sara Lopez-Gomollon, PhD, Guest Editor

Pablo Manavella, Editor-in-Chief

The Plant Cell

Response: We have conducted additional experiments and revised the manuscript to

improve its clarity and scientific quality. Furthermore, we have replaced the original red/green contrast of RFP and GFP fluorescence signals with pseudo-colored magenta/green in all figures, for readers with color vision deficiencies.

Reviewers' comments:

Reviewer 1: This article provides a thorough and well-founded analysis of how the m^6A modification of viral RNA contributes to RNA stability and viral infection by barley yellow striate mosaic virus (BYSMV), and how plants counteract this process through the demethylase HvALKBH1B. Through rigorous experiments, it is shown that m^6A modification in the viral *P6* mRNA is crucial for RNA stability and virus infectivity. Additionally, HvALKBH1B is identified as an antiviral defense by removing m^6A from viral RNA. This study not only offers new perspectives on understanding plant and virus biology and post-transcriptional regulation, but also opens the door to genetic improvement strategies for growing virus-resistant plants.

The work is well-executed and the article is well-written, although some conclusions do not appear to be fully supported by the data obtained. While the level of detail and rigor in the experiments is recognized, there are several points that require further discussion and adjustments in the presentation of results (major objections):

Response: We sincerely thank you for your recognition and valuable suggestions. We have performed additional experiments and revised the manuscript to improve the logic and strengthen the results and conclusions as following.

* Alternative methods to MeRIP-seq and m^6A antibodies: In line 136, the authors mention using MeRIP-seq to examine the m^6A modification in the BYSMV viral genome. However, the specificity of the m^6A antibody used in this experiment is questioned. It would be prudent to include positive and negative controls in these experiments, as well as discuss whether alternative methods, such as Nanopore sequencing, could be useful to confirm results without relying on antibodies (which have a higher false-positive rate).

Response: Many thanks for your positive comments and constructive suggestions. We evaluated Nanopore sequencing as an alternative approach for detecting m^6A modifications in BYSMV. However, this technique is not yet widely available in China, and current providers cannot guarantee the generation of high-quality data for analysis of viral RNA derived from plant hosts.

MeRIP-seq is one of the most widely used techniques for identifying m^6A modifications on RNA. When combined with m^6A-IP-qPCR, it allows for relatively

accurate detection of m^{6}A modifications on specific RNA transcripts. This approach has been successfully applied in several studies to detect m^{6}A modifications on the RNA of both animal and plant viruses (Lichinchi et al. 2016; Zhang et al. 2022). To validate the specificity of the antibody used, we synthesized two RNA fragments: one containing m^{6}A-modified *P6* RNA and another unmodified *P6* RNA as a control. Dot blot assays confirmed that the antibody specifically recognizes m^{6}A-modified RNA (see Figure below).

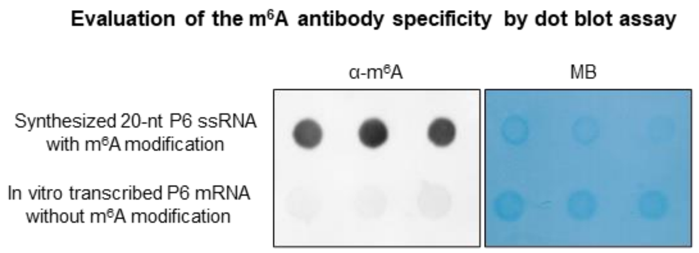

In our revised m^{6}A-IP experiments (Figure 1B), the *RFP* mRNA from BYSMV-RFP virus was used as a negative control. The results showed that only the *P6* mRNA was highly enriched, indicating specific m^{6}A modification. Furthermore, the SELECT assay (single-base elongation- and ligation-based qPCR amplification method) presented in our original Figure 1C further confirmed the presence of m^{6}A modifications on *P6* mRNA. Together, these results provide strong evidence that BYSMV *P6* mRNA contains m^{6}A modifications in host plants.

Reference:

(1) Lichinchi, G., Zhao, B.S., Wu, Y., Lu, Z., Qin, Y., He, C., and Rana, T.M. (2016). Dynamics of human and viral RNA methylation during zika virus infection. Cell Host & Microbe 20, 666-673.

(2) Zhang, T., Shi, C., Hu, H., Zhang, Z., Wang, Z., Chen, Z., Feng, H., Liu, P., Guo, J., Lu, Q., Zhong, K., Chen, Z., Liu, J., Yu, J., Chen, J., Chen, F., and Yang, J. (2022). N^6-methyladenosine RNA modification promotes viral genomic RNA stability and infection. Nat. Commun. 13, 6576.

* Statistical analysis of fluorescent signals: In several sections of the article, such as lines 163 and 176-177, differences in fluorescent signal intensity are mentioned, but no proper statistical analysis is provided. The claim that the signal is "significantly reduced" should be supported by quantitative measurements and statistical analysis to avoid ambiguous interpretations of the data.

Response: Many thanks for your constructive suggestions. We performed quantitative

analysis of the fluorescence intensity in the indicated figures using ImageJ software, and conducted statistical analysis of significance. Furthermore, in BYSMV-infected *N. benthamiana*, we counted the number of infected cells based on the RFP signal. All statistical results are presented as histograms in the main or supplementary figures. Please see Figure 1E, 1K, Supplementary Fig. S3B, S3G, S6B, S7C, S9B, and S10E.

* Analysis of IDR mutants and protein stability: In line 283, the authors discuss mutants with deletions in the IDR region of HvALKBH1B to study its role in phase separation. However, deletions in large IDR regions (50-150 aminoacids) could affect protein stability and function. A more detailed aminoacid-level analysis (e.g., tyrosines, glycines, and arginines, which are widely known for their role in phase separation) or domain analysis would be helpful to understand how these mutations affect phase separation and antiviral function.

Response: Many thanks for your constructive suggestions. We examined the protein stability of the HvALKBH1B$^{\Delta IDR3}$ mutant. Immunoblot analysis showed that HvALKBH1B$^{\Delta IDR3}$ even accumulated at a higher level than the full-length HvALKBH1B. Please see Supplementary Fig. S8.

To dissect the minimal motif within the IDR3 that is essential for the phase separation of HvALKBH1B, we have constructed several truncated mutants in the IDR3 region of the full-length protein. A C-terminal 38-amino-acid deletion (HvALKBH1B$^{\Delta C38}$) lost the LLPS activity. To further define the critical residues, we substituted ten serine and arginine residues within this region with alanine (HvALKBH1B$^{RS/AT}$), which was completely abolished the ability of LLPS. We next constructed recombinant BYSMV vectors to overexpress HvALKBH1B$^{\Delta C38}$ and HvALKBH1B$^{RS/AT}$ in *N. benthamiana* to assess their antiviral activity. Consistent with the HvALKBH1B$^{\Delta IDR3}$ mutant, neither HvALKBH1B$^{\Delta C38}$ or HvALKBH1B$^{RS/AT}$ suppressed BYSMV infection. These results indicate that the phase separation capacity of HvALKBH1B is essential for its potent antiviral activity. Please see Supplementary Fig. S10.

* Overexpression of a truncated version of HvALKBH1B: In line 318, the authors overexpress a truncated version of HvALKBH1B (HvALKBH1B^{T}), which lacks the N-terminal region. Since this truncated version retains the ability to form granules, but could affect other protein functions, it is suggested to overexpress the full-length protein to gain a more accurate understanding of HvALKBH1B's impact on BYSMV infection. Additionally, in the abstract and discussion (line 412), the authors mention that the overexpression of HvALKBH1B confers strong resistance to BYSMV. However, since a truncated version of the protein is used in experiments, it would be relevant to clarify

this detail in both sections to avoid confusion.

Response: Many thanks for your suggestions. When we planned to obtain overexpression transgenic plants 3 years ago, the barley reference genome only annotated the truncated isoform *HvALKBH1B*^{T} (*HORVU7Hr1G018340*). Recently, a more accurate genome assembly revealed the full-length *HvALKBH1B* sequence (*HORVU.MOREX.r3.7HG0647650*). Both versions of the gene are available in the Plant Genomics Resource database (https://phytozome-next.jgi.doe.gov/). Considering the time and resource constraints associated with generating new transgenic lines de novo. We analysed the HvALKBH1B^{T} with respect to its subcellular localization, phase separation behavior, and antiviral activity, and found no obvious differences compared with HvALKBH1B (Please see Supplementary Fig. S11). Additionally, we generated CRISPR-Cas9-mediated knockout lines of *HvALKBH1B*, which further confirmed its essential role in restricting BYSMV infection.

In the revised manuscript, we have clarified in both the Abstract and Discussion sections that the overexpression transgenic lines used in this study express a truncated version of HvALKBH1B (HvALKBH1B^{T}).

There are several minor issues that do not greatly affect the discussion and presentation of conclusions. These corrections would be timely for a more coherent and concise presentation of the results (minor objections):

* Clarity of technical terms: In line 61, the term "RNA antigenome" is used to refer to m^{6}A methylation on the negative strand of BYSMV. This term is not commonly used in the scientific literature, so it would be more appropriate to refer simply to the "negative RNA strand." This change would clarify the description and avoid confusion for readers.

Response: Line 61 refers to the role of m^{6}A modification in the genome and antigenome of human metapneumovirus (HMPV), as described in the cited reference (Lu et al., 2020). Similar to BYSMV, HMPV is a negative-strand RNA virus, whose genome is a negative strand RNA. The antigenome, is the positive-strand RNA that serves as the template for synthesizing new viral genomes during replication.

Reference: Lu, M., Zhang, Z., Xue, M., Zhao, B.S., Harder, O., Li, A., Liang, X., Gao, T.Z., Xu, Y., Zhou, J., Feng, Z., Niewiesk, S., Peeples, M.E., He, C., and Li, J. (2020). N^{6}-methyladenosine modification enables viral RNA to escape recognition by RNA sensor RIG-I. Nature Microbiology 5, 584-598.

* P6 mutants and modifications at methylation sites: In line 150, the m^{6}A modification

sites in P6 mRNA are identified, highlighting significant methylation peaks at A53, A122, A174, and A262. However, in constructing the $P6^{4m}$-GFP quadruple mutant, the authors mutate a relatively large number of adenosines. It would be more interesting to mutate a smaller number of sites, especially those with significant p-values (A53 and A122), to avoid excessive alteration of the P6 protein's structure.

Response: Many thanks for your suggestions. We have performed an additional experiment to construct a double mutant at A53 and A122 of the P6 gene ($P6^{A53/122T}$), which also disrupts the stability of *P6* mRNA. Consistent with this, the BYSMV mutation of $P6^{A53/122T}$ exhibits attenuated infectivity compared with the wild-type virus. Please see Supplementary Fig. S3.

* Selection of demethylases for the study: In line 200, the authors choose to focus on the demethylase HvALKBH1B due to its relationship with AtALKBH9B, but do not discuss in detail the potential effects of other cytoplasmic demethylases, such as HvALKBH2B or HvALKBH7B, which could play a role in BYSMV infection. It would be interesting to briefly explore these possible roles and discuss whether these proteins might have a redundant or proviral function similar to AtALKBH9B in other viruses.

Response: Many thanks for your suggestions. In the revised manuscript, we used the BYSMV vector to overexpress two other cytoplasmic demethylases, HvALKBH2B and HvALKBH7B, to compared with HvALKBH1B. Neither HvALKBH2B nor HvALKBH7B inhibited BYSMV infection. Please see Supplementary Fig. S7.

* Phase condensates and in vitro/in vivo results: In the paragraphs of lines 242 and 258, the authors show that HvALKBH1B forms phase condensates both in vitro and in vivo. It would be more logical to present the in vitro experiments first, followed by the in vivo results, which would improve the article's narrative coherence.

Response: Many thanks for your suggestions. We have revised the manuscript by reordering the relevant statements to improve the logical structure of the text. Please see lines 261-279.

* Units: The absence of relative units on the y-axis (0-25) in Figure 1A makes it difficult to interpret the methylation peaks across the BYSMV genome. Adding units would improve clarity.

Response: Many thanks for your suggestions. Figure 1 shows the normalization analysis of the sequencing results, visualized using IGV software. The numbers on the y-axis indicate the range of coverage values. These details have been labeled in the

revised Figures and are also mentioned in the updated Figure legends.

In summary, this work makes a valuable contribution to the study of the role of HvALKBH1B in viral infection, but the conclusions are not sufficiently supported by the experimental data presented. Significant adjustments are needed in both methodology and data interpretation to strengthen the claims made in the article.

Response: Thank you again for your recognition and valuable suggestions. We have performed additional experiments and revised the manuscript according to your comments in order to improve its quality.

Reviewer 2: RNA m^{6}A modification plays a crucial role in plant responses to both biotic and abiotic stresses. However, studies focusing on negative-strand RNA viruses and barley plant remain limited. This manuscript by Zang et al. investigates an insect-transmitted plant rhabdovirus (BYSMV) and reveals that m6A modification of viral RNA contributes to its stabilization and accumulation, thereby facilitating viral infection. The authors further identified a barley m^{6}A demethylase, HvALKBH1B, that undergoes liquid-liquid phase separation (LLPS) and mediates antiviral defense. Overall, the manuscript is clearly written, concise, and provides new insights into the role of m6A in plant-virus interactions. This manuscript is very interesting and a good fit to Plant cell. I only have very minor suggestions the authors may consider:

Response: We sincerely thank you for your positive and constructive comments, and for your time and effort in reviewing our manuscript.

1. As alkbh mutants have been reported to cause male sterility in rice (Tang et al., 2024) and affect fruit ripening in tomato (Zhou et al., 2025). What about the role of the alkbh1b mutant in barley development? The authors only showed some barley seedlings in Figure S7.

Response: Many thanks for your suggestions. In the revised materials, we presented the 40-day and 140-day growth phenotypes of *hvalkbh1b* barley. No visible differences were observed compared with the wild type, and the mutant plants produced normal seeds. Please see Supplementary Fig. S12.

2. Since BYSMV is transmitted by insects and replicates within their bodies, an open question is whether the viral *P6* mRNA is subject to m^{6}A modification in the insect.

Response: We performed m^{6}A-IP-qPCR using RNA extracted from BYSMV-RFP–infected small brown planthoppers. The results showed that *P6* mRNA is highly modified with m^{6}A (see Figure below). Our previous study (Gao et al., 2023) demonstrated that the viral P6 protein modulates the locomotor activity of insect vectors.

These results suggest that BYSMV may utilize m^{6}A modification to enhance the accumulation of P6 in insect, which may be required for its behavioral manipulation of the vectors.

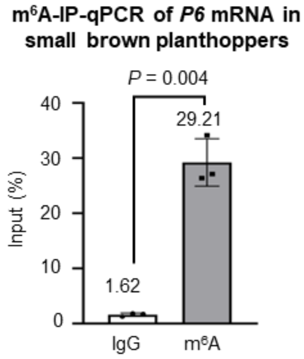

Reference: Gao, D.M., Qiao, J.H., Gao, Q., Zhang, J., Zang, Y., Xie, L., Zhang, Y., Wang, Y., Fu, J., Zhang, H., Han, C., and Wang, X.B. (2023). A plant cytorhabdovirus modulates locomotor activity of insect vectors to enhance virus transmission. Nat. Commun. 14, 5754.

3. Line 188 "BYSMV exploits m^{6}A RNA modification to stable" should be "to stabilize".

Response: Thanks! We have corrected it. Please see line 198.

4. Line 356 "the resistance against BYSMV are abolished" should be "is abolished".

Response: Thanks! We have corrected it. Please see line 381.

5. Line 414-416, the description may not be appropriate, as wheat yellow mosaic virus (WYMV) is a positive-sense RNA virus that requires m^{6}A modification to facilitate infection.

Response: Thanks for your suggestions. We have changed the description. Please see lines 439-440.

6. Line 566 "2 L-ascorbic acid" is missing a unit.

Response: Thanks! We have corrected it. Please see lines 601.

7. Line 657. "with emissions captured at 600-630 nm,630 nm", the 630 nm may be unnecessary.

Response: Thanks! We have corrected it. Please see lines 692.

In compliance with data protection regulations, you may request that we remove your personal registration details at any time. (Use the following URL: https://www.editorialmanager.com/tpc/login.asp?a=r). Please contact the publication office if you have any questions.

**Date:**	Apr 10, 2025
**To:**	"qiang gao" qianggao@cau.edu.cn
**cc:**	"Ying Zang" zb20193020217@cau.edu.cn, "Ji-Hui Qiao" sz20183020170@cau.edu.cn, "De-Shui Liu" deshui@yeah.net, "Dong-Min Gao" 85012885@qq.com, "Xin-Wen Zhang" sz20213020221@cau.edu.cn, "Tian-Tian Pan" zb20243020259@cau.edu.cn, "Yi-Zhou Yang" yizhou.yang@qq.com, "Xian-Bing Wang" wangxianbing@cau.edu.cn
**From:**	"The Plant Cell" tpc-submissions@aspb.org
**Subject:**	TPC-2025-0116 Decision Letter

Ref.: Ms. No. TPC-2025-0116
Barley m6A demethylase HvALKBH1B undergoes phase separation to enhance antiviral immunity against a plant rhabdovirus
The Plant Cell

Dear gao,

We have received reviews of the manuscript above. Thank you for submitting your best work to The Plant Cell. The editorial board agrees that the work you describe is substantive, falls within the scope of the journal, and may become acceptable for publication, pending revision and potential re-review.
Please see the reviewer comments below for specific items to address in your next draft. Do not hesitate to contact us if you have questions about any of the requested revisions.

A note from the Editor-in-Chief: We are trying to make a concerted effort to change green/red comparisons to green/magenta to make our figures understandable to those with color vision deficiencies. We noticed that a small number of your figures (for example Fig. 2A, 4K, 5E) utilize red/green contrasts, so as you prepare the final version of the figures, please check the figures for red/green color use. Magenta is a good substitute for red, and a light blue substitutes for green even if the data are labeled as "RFP" or "mCherry" - readers will understand that colors can be changed, and indeed, it can be adjusted relatively quickly by using the "hue" setting. You might want to check over the Supplemental Figures for similar comparisons. Please note that color changes do not need to be highlighted or tracked in the revised manuscript, but could be noted in the cover letter or response document.

Given the nature of the comments, we are offering you 60 days from when we have issued this decision to complete the revision. If a revision is not returned within this time frame, and if you have not been granted an extension, we will withdraw the manuscript, which will leave you free to submit the work elsewhere. If you need an extension, we encourage you to contact us at any point before the 60 days have passed.

When you are ready to submit the revised version, please upload a highlighted copy that indicates all changes made in response to the editor and reviewer recommendations. Include an itemized list of all changes made in response to each of the reviewer's suggestions in the "Response to Reviewers" section; please note that reviewers do not have access to your cover letter, nor was this decision letter shared with them.

Thank you for the privilege of reviewing your work. We look forward to receiving your revised manuscript.

Yours sincerely

Sara Lopez-Gomollon, PhD, Guest Editor
Pablo Manavella, Editor-in-Chief
The Plant Cell

Reviewers' comments:
Reviewer 1: This article provides a thorough and well-founded analysis of how the m6A modification of viral RNA contributes to RNA stability and viral infection by barley yellow striate mosaic virus (BYSMV), and how plants counteract this process through the demethylase HvALKBH1B. Through rigorous experiments, it is shown that m6A modification in the viral P6 mRNA is crucial for RNA stability and virus infectivity. Additionally, HvALKBH1B is identified as an antiviral defense by removing m6A from viral RNA. This study not only offers new perspectives on understanding plant and virus biology and post-transcriptional regulation, but also opens the door to genetic improvement strategies for growing virus-resistant plants.
The work is well-executed and the article is well-written, although some conclusions do not appear to be fully supported by the data obtained. While the level of detail and rigor in the experiments is recognized, there are several points that require further discussion and adjustments in the presentation of results (major objections):
* Alternative methods to MeRIP-seq and m6A antibodies: In line 136, the authors mention using MeRIP-seq to examine the m6A modification in the BYSMV viral genome. However, the specificity of the m6A antibody used in this experiment is questioned. It would be prudent to include positive and negative controls in these experiments, as well as discuss whether alternative methods, such as Nanopore sequencing, could be useful to confirm results without relying on antibodies (which

have a higher false-positive rate).
* Statistical analysis of fluorescent signals: In several sections of the article, such as lines 163 and 176-177, differences in fluorescent signal intensity are mentioned, but no proper statistical analysis is provided. The claim that the signal is "significantly reduced" should be supported by quantitative measurements and statistical analysis to avoid ambiguous interpretations of the data.
* Analysis of IDR mutants and protein stability: In line 283, the authors discuss mutants with deletions in the IDR region of HvALKBH1B to study its role in phase separation. However, deletions in large IDR regions (50-150 aminoacids) could affect protein stability and function. A more detailed aminoacid-level analysis (e.g., tyrosines, glycines, and arginines, which are widely known for their role in phase separation) or domain analysis would be helpful to understand how these mutations affect phase separation and antiviral function.
* Overexpression of a truncated version of HvALKBH1B: In line 318, the authors overexpress a truncated version of HvALKBH1B (HvALKBH1BT), which lacks the N-terminal region. Since this truncated version retains the ability to form granules, but could affect other protein functions, it is suggested to overexpress the full-length protein to gain a more accurate understanding of HvALKBH1B's impact on BYSMV infection. Additionally, in the abstract and discussion (line 412), the authors mention that the overexpression of HvALKBH1B confers strong resistance to BYSMV. However, since a truncated version of the protein is used in experiments, it would be relevant to clarify this detail in both sections to avoid confusion.

There are several minor issues that do not greatly affect the discussion and presentation of conclusions. These corrections would be timely for a more coherent and concise presentation of the results (minor objections):
* Clarity of technical terms: In line 61, the term "RNA antigenome" is used to refer to m6A methylation on the negative strand of BYSMV. This term is not commonly used in the scientific literature, so it would be more appropriate to refer simply to the "negative RNA strand." This change would clarify the description and avoid confusion for readers.
* P6 mutants and modifications at methylation sites: In line 150, the m6A modification sites in P6 mRNA are identified, highlighting significant methylation peaks at A53, A122, A174, and A262. However, in constructing the P64m-GFP quadruple mutant, the authors mutate a relatively large number of adenosines. It would be more interesting to mutate a smaller number of sites, especially those with significant p-values (A53 and A122), to avoid excessive alteration of the P6 protein's structure.
* Selection of demethylases for the study: In line 200, the authors choose to focus on the demethylase HvALKBH1B due to its relationship with AtALKBH9B, but do not discuss in detail the potential effects of other cytoplasmic demethylases, such as HvALKBH2B or HvALKBH7B, which could play a role in BYSMV infection. It would be interesting to briefly explore these possible roles and discuss whether these proteins might have a redundant or proviral function similar to AtALKBH9B in other viruses.
* Phase condensates and in vitro/in vivo results: In the paragraphs of lines 242 and 258, the authors show that HvALKBH1B forms phase condensates both in vitro and in vivo. It would be more logical to present the in vitro experiments first, followed by the in vivo results, which would improve the article's narrative coherence.
* Units: The absence of relative units on the y-axis (0-25) in Figure 1A makes it difficult to interpret the methylation peaks across the BYSMV genome. Adding units would improve clarity.

In summary, this work makes a valuable contribution to the study of the role of HvALKBH1B in viral infection, but the conclusions are not sufficiently supported by the experimental data presented. Significant adjustments are needed in both methodology and data interpretation to strengthen the claims made in the article.

Reviewer 2: RNA m6A modification plays a crucial role in plant responses to both biotic and abiotic stresses. However, studies focusing on negative-strand RNA viruses and barley plant remain limited. This manuscript by Zang et al. investigates an insect-transmitted plant rhabdovirus (BYSMV) and reveals that m6A modification of viral RNA contributes to its stabilization and accumulation, thereby facilitating viral infection. The authors further identified a barley m6A demethylase, HvALKBH1B, that undergoes liquid-liquid phase separation (LLPS) and mediates antiviral defense. Overall, the manuscript is clearly written, concise, and provides new insights into the role of m6A in plant-virus interactions. This manuscript is very interesting and a good fit to Plant cell. I only have very minor suggestions the authors may consider:

1. As alkbh mutants have been reported to cause male sterility in rice (Tang et al., 2024) and affect fruit ripening in tomato (Zhou et al., 2025). What about the role of the alkbh1b mutant in barley development? The authors only showed some barley seedlings in Figure S7.
2. Since BYSMV is transmitted by insects and replicates within their bodies, an open question is whether the viral P6 mRNA is subject to m6A modification in the insect.
3. Line 188 "BYSMV exploits m6A RNA modification to stable" should be "to stabilize".
4. Line 356 "the resistance against BYSMV are abolished" should be "is abolished".
5. Line 414-416, the description may not be appropriate, as wheat yellow mosaic virus (WYMV) is a positive-sense RNA virus that requires m6A modification to facilitate infection.
6. Line 566 "2 L-ascorbic acid" is missing a unit.
7. Line 657. "with emissions captured at 600-630 nm,630 nm", the 630 nm may be unnecessary.

In compliance with data protection regulations, you may request that we remove your personal registration details at any time. (Use the following URL: https://www.editorialmanager.com/tpc/login.asp?a=r). Please contact the publication office if you have any questions.
